# Effectiveness of Telerehabilitation for Chronic Nonspecific Low Back Pain: Systematic Review and Meta-Analysis of Randomized Controlled Trials

**DOI:** 10.2196/94835

**Published:** 2026-08-10

**Authors:** Hongyuan Wang, Zhixiang Liu, Yan Li, Shufang Li, Meng Gong, Lina Pang, Xiangyin Ye, Song Jin

**Affiliations:** 1 School of Health Preservation and Rehabilitation, Chengdu University of Traditional Chinese Medicine, Chengdu, Sichuan, China; 2 Department of Rehabilitation, The Third People's Hospital of Bengbu, Bengbu, Anhui, China; 3 School of Acupuncture and Tuina, Chengdu University of Traditional Chinese Medicine, Chengdu, Sichuan, China; 4 Department of Rehabilitation, Hospital of Chengdu University of Traditional Chinese Medicine, 39 Shierqiao Road, Jinniu District, Chengdu, Sichuan, China, 86 18980880353

**Keywords:** chronic nonspecific low back pain, telerehabilitation, systematic review, meta-analysis, digital health

## Abstract

**Background:**

Chronic nonspecific low back pain (CNSLBP) is one of the most prevalent pain disorders worldwide. Telerehabilitation is increasingly used in the management of CNSLBP. However, evidence on its effects in CNSLBP remains inconsistent.

**Objective:**

This systematic review and meta-analysis aimed to evaluate the potential effects of telerehabilitation on pain intensity, functional disability, and kinesiophobia in adults with CNSLBP and to explore whether these effects differed by comparator intensity, delivery mode, and supervision level.

**Methods:**

PubMed, Embase, Web of Science, PEDro, Cochrane Central Register of Controlled Trials, China National Knowledge Infrastructure, Wanfang, VIP, and China Biology Medicine were searched from inception to May 8, 2026. Randomized controlled trials evaluating telerehabilitation in adults with CNSLBP were included. Risk of bias was assessed using the Cochrane Risk of Bias 2.0 tool, and the certainty of evidence was evaluated using the Grading of Recommendations Assessment, Development, and Evaluation approach. Random-effects meta-analyses were performed using the Hartung-Knapp-Sidik-Jonkman adjustment, and 95% prediction intervals (PIs) were calculated using the Nagashima confidence distribution approach.

**Results:**

A total of 14 randomized controlled trials involving 794 participants were included. Overall, telerehabilitation was associated with statistically significant average reductions in pain intensity (mean difference [MD] −1.02, 95% CI −1.78 to −0.26; *P*=.01; *I²*=89.9%; 95% PI −3.61 to 1.51) and functional disability (MD −7.04, 95% CI −13.43 to −0.65; *P*=.03; *I²*=82.6%; 95% PI −27.97 to 13.91) compared with control conditions, whereas the overall effect on kinesiophobia was not statistically significant (MD −3.14, 95% CI −7.88 to 1.59; *P*=.15; *I²*=86.5%; 95% PI −15.04 to 8.47). All 95% PIs crossed the null, indicating substantial uncertainty in the expected effects across clinical settings. Subgroup analyses suggested that average benefits were mainly observed when telerehabilitation was compared with minimal or nonactive controls. Compared with active controls, telerehabilitation showed no clear advantage for pain or kinesiophobia, and functional disability slightly favored active rehabilitation. Exploratory subgroup analyses by delivery mode and supervision level suggested possible differences across intervention models, but these findings should not be interpreted causally because several subgroups included few studies and these factors were not mutually independent.

**Conclusions:**

This review highlights the importance of comparator intensity, delivery mode, and supervision level when interpreting telerehabilitation effects. Telerehabilitation may provide context-dependent benefits, particularly when compared with minimal or nonactive management, but current evidence does not show a clear advantage over structured active rehabilitation. Given the substantial heterogeneity, PIs crossing the null, risk of bias, and low or very low certainty of evidence, telerehabilitation may be better positioned as a flexible or adjunctive approach to improve access to and continuity of care rather than as a replacement for active rehabilitation. Future trials should use standardized comparators, improve intervention reporting, include longer follow-up, and evaluate implementation and cost-effectiveness.

## Introduction

Low back pain (LBP) is a major contributor to the global burden of disease and one of the leading causes of years lived with disability, being strongly associated with substantial functional limitation [1,2]. Data from the Global Burden of Disease study indicate that the number of individuals affected by LBP continues to rise and is projected to reach 843 million by 2050 [3]. In most patients, symptoms cannot be clearly attributed to a specific pathoanatomical abnormality and are therefore classified as nonspecific LBP [4], which accounts for approximately 90% to 95% of all LBP cases [5,6]. When pain and functional limitations persist for at least 3 months, the condition is defined as chronic nonspecific low back pain (CNSLBP) [7]. CNSLBP may result not only in persistent or recurrent pain but also in functional disability, activity limitation, emotional distress, absenteeism, and reduced work capacity [8-10].

CNSLBP is characterized by a prolonged clinical course, a high risk of recurrence, and multifactorial involvement [11,12]. Accordingly, current clinical practice guidelines generally emphasize health education and self-management support as the foundation of care, with exercise therapy recommended as a first-line intervention. When necessary, psychological interventions and other nonpharmacological treatments may also be integrated by addressing pain-related beliefs, fear-avoidance behaviors, emotional distress, and other psychosocial factors, with the aim of promoting activity restoration and functional improvement [13-17]. However, traditional face-to-face rehabilitation usually requires repeated in-person visits, which are often limited by scheduling difficulties, travel distance, and financial burden. As a result, some patients may have difficulty accessing continuous, standardized rehabilitation management and regular follow-up [18,19]. In addition, in the absence of ongoing supervision and individualized guidance, the effectiveness of home-based exercise and self-management is often limited [20].

Telerehabilitation is commonly defined as the delivery, support, or monitoring of rehabilitation services through information and communication technologies, including mobile apps, web-based platforms, video-based programs, virtual reality systems, telephone support, and videoconferencing [21]. By reducing the need for repeated in-person visits, telerehabilitation may improve access to rehabilitation services, support continuity of care, and reduce travel- and time-related burdens [22-24].

In this review, telerehabilitation was conceptualized as a mode of rehabilitation delivery rather than as a single therapeutic intervention or a broad digital health category. Digital or mobile health tools were considered telerehabilitation only when they were used to deliver, guide, support, monitor, or provide feedback on rehabilitation-oriented interventions for CNSLBP, such as therapeutic exercise, rehabilitation-related education, self-management support, adherence monitoring, or individualized feedback. In contrast, tools limited to general health information, passive data collection, or remote monitoring without a rehabilitation-related therapeutic component were not considered telerehabilitation. This conceptual boundary is important because telerehabilitation models may differ substantially in delivery mode, therapist involvement, supervision intensity, feedback mechanisms, adherence support, and the extent to which digital tools are used for active rehabilitation delivery rather than for passive monitoring or general support alone [25].

Although previous systematic reviews have evaluated eHealth, mobile health, or telerehabilitation interventions for LBP [22,26-29], the applicability of this evidence to CNSLBP remains limited by several important gaps. First, at the population level, some previous reviews included broader or more heterogeneous LBP populations and did not consistently distinguish CNSLBP from other LBP conditions, which may limit the direct applicability of their findings to adults with CNSLBP [29]. Second, the conclusions of existing reviews have not been entirely consistent. Some reviews suggested that remote interventions may improve pain and function in the short term, particularly when used as an adjunct to usual care [26,28,29], whereas others found no clear superiority over usual care or face-to-face rehabilitation or suggested that telerehabilitation achieved broadly comparable effects [22,27]. In addition, previous syntheses have not fully clarified how delivery mode, supervision level, feedback or monitoring methods, and comparator intensity may influence treatment effects, further limiting the clinical interpretation of the evidence. Among these factors, differences in comparator intensity deserve particular attention. Control conditions have varied substantially across trials, which may be an important reason for the inconsistent findings reported in previous reviews.

Clarifying these issues is important for interpreting how telerehabilitation should be used in CNSLBP care. Therefore, this systematic review and meta-analysis aimed to (1) evaluate the effects of telerehabilitation on pain intensity, functional disability, and kinesiophobia in adults with CNSLBP; and (2) explore whether these effects differed according to comparator intensity, delivery mode, and supervision level.

## Methods

### Study Design and Reporting Guidelines

This systematic review was conducted in accordance with the PRISMA (Preferred Reporting Items for Systematic Reviews and Meta-Analyses) guidelines [30], using the PRISMA 2020 checklist (Checklist 1).

### Registration and Protocol Deviations

This review was registered in PROSPERO under registration number CRD420251151727. The original record was made available on September 20, 2025, and an amended record focusing on telerehabilitation for CNSLBP was made available on January 10, 2026. As the registration record was substantially amended from cognitive functional therapy to telerehabilitation, with changes in the review question, intervention definition, eligibility criteria, search sources, outcomes, and synthesis plan, this review is reported as a registered review with a subsequent major protocol amendment rather than as a fully prospective registration of the final review question. The main protocol deviations and corresponding corrective actions are reported in Multimedia Appendix 1.

### Eligibility Criteria

Eligibility criteria were defined according to the population, intervention, comparator, outcomes, and study design (PICOS) framework (Textbox 1).

Textbox 1. **Eligibility criteria based on the population, intervention, comparator, outcomes, and study design framework.**

Inclusion criteria

- Participants: Adults with chronic nonspecific low back pain were eligible. Chronicity was defined as low back pain lasting at least 12 wk or 3 mo. Studies using the broader term “chronic low back pain” were considered eligible only when participants had no radicular symptoms or when serious or specific spinal pathology, such as malignancy, recent spinal surgery, pregnancy-related low back pain, inflammatory disease, fracture, spinal stenosis, spondylolisthesis, or predominant radicular pain, was excluded.
- Intervention: Telerehabilitation was defined as the remote delivery or support of rehabilitation-related interventions through digital or communication technologies, including web-based platforms, mobile apps, video-based programs, virtual reality systems, telephone or video call support, and hybrid models combining initial in-person instruction with remote follow-up. We considered telerehabilitation as a mode of care delivery rather than a single therapeutic intervention.
- Comparison: (1) Active controls: therapist-led or therapist-supervised structured face-to-face rehabilitation, such as outpatient physical therapy or face-to-face supervised structured exercise training; (2) minimal or nonactive controls: wait list or no treatment, maintenance of usual activities, education or general advice, unsupervised home exercise, or self-management without therapist supervision (including interventions limited to written materials or nonindividualized guidance); (3) when the comparator was described as “usual care” or consisted of complex mixed interventions, it was classified as either an active control or a minimal or nonactive control based on the actual treatment components received by the control group, the intensity of supervision, and the degree of individualization reported in the trial. The classification was performed independently by 2 reviewers (HW and YL), and disagreements were resolved by a third reviewer (SL).
- Outcomes: At least one of the following outcomes was reported: pain intensity (visual analog scale and Numerical Rating Scale), functional disability (Oswestry Disability Index), or kinesiophobia (Tampa Scale of Kinesiophobia);
- Study design: randomized controlled trials.
- Language: studies published in English or Chinese were eligible.

Exclusion criteria

- Paper type: reviews, conference abstracts, study protocols, and case reports
- Interventions: interventions that did not fall within the scope of telerehabilitation
- Outcomes: studies in which key outcome data could not be extracted or pooled

### Search Strategy

The systematic literature search was designed, conducted, and reported in accordance with the PRISMA-S (Preferred Reporting Items for Systematic Reviews and Meta-Analyses—Search Extension) guidelines [31]. The following electronic databases were systematically searched from inception to November 2, 2025, with a final update on May 8, 2026: PubMed (via National Center for Biotechnology Information), Embase (via Elsevier), Web of Science (via Clarivate), PEDro, Cochrane Central Register of Controlled Trials (via Wiley), China National Knowledge Infrastructure, Wanfang, VIP, and China Biology Medicine (via SinoMed). All databases were searched individually via their native platforms; no multidatabase platform searching was used. The search strategy generally combined MeSH terms, EMTREE terms where applicable, and free-text terms related to LBP, telerehabilitation, telemedicine, digital health, and randomized controlled trials (RCTs). RCT-related search terms were used to improve search specificity; no published validated search filter was used. The search strategies were developed de novo for this review and were not adapted from prior systematic reviews or previously published searches. The search strategies were adapted to the syntax, indexing structure, and search interface of each database. No restrictions on publication year were applied. Studies published in English or Chinese were eligible. The reference lists of all eligible studies were manually searched to identify additional relevant studies. No other online resources or study registries were searched. No study authors, domain experts, or other individuals were contacted to identify additional studies or obtain supplementary data. The search strategies were developed and internally checked by the research team but did not undergo external peer review. The complete search strategies for all databases are provided in Multimedia Appendix 2.

### Selection Process

All retrieved records were imported into EndNote 20 (Clarivate) for reference management and duplicate removal. Duplicate records were first removed using the software’s automated duplicate detection function and then manually checked by the reviewers to identify any remaining duplicates. After deduplication, 2 reviewers (HW and YL) independently screened the titles and abstracts of all records according to the predefined eligibility criteria. Potentially eligible studies were then assessed through full-text review. Disagreements between the 2 reviewers were resolved through discussion or consultation with a third reviewer (SL).

### Outcome Measures

The outcomes of interest in this study included pain intensity, kinesiophobia, and functional disability. Pain intensity was assessed using the visual analog scale and the Numerical Rating Scale, both of which are widely used tools for pain assessment in clinical practice and research and have demonstrated good reliability and validity [32,33]. Functional disability was evaluated using the Oswestry Disability Index [34]. Kinesiophobia was assessed using the Tampa Scale of Kinesiophobia, which is commonly used in populations with LBP to reflect fear of movement and related avoidance behaviors [35].

### Data Extraction

Two reviewers (HW and YL) independently extracted the main information from the included studies using a standardized data extraction form created in Excel (Microsoft Corp). In cases of disagreement between the 2 reviewers, a third reviewer (SL) was consulted for adjudication. The extracted information included general study characteristics (author, year of publication, country, and sample size), participant characteristics (sex and age), intervention characteristics, comparator characteristics, and outcome measures (pain intensity, kinesiophobia, and functional disability). For intervention characterization, we extracted the telerehabilitation modality, delivery mode, intervention components, duration, and frequency, supervision level, and feedback or monitoring methods. Comparator intensity was classified as active control or minimal or nonactive control based on the comparator definitions specified in the eligibility criteria.

For intervention characterization and exploratory subgroup analyses, supervision level was operationally classified as low, moderate, or high according to the degree, timing, and individualization of therapist involvement. Classification was performed independently by 2 reviewers (HW and YL), with disagreements resolved by a third reviewer (SL). Low supervision indicated self-directed or automated interventions without scheduled individualized therapist contact or feedback. Moderate supervision indicated scheduled asynchronous or periodic therapist contact, remote monitoring, adherence support, or individualized non–real-time feedback, but without real-time therapist supervision of each treatment session. High supervision indicated synchronous therapist-led sessions with real-time guidance, exercise correction, and individualized feedback during the intervention session. When an intervention included features across more than one category, classification was based on the highest level of therapist interaction that was consistently delivered during the intervention period.

If multiple follow-up time points were reported in an included study, data assessed immediately after the intervention (post-intervention) were preferentially extracted and pooled. When data were reported as medians and IQRs, they were converted into means and SDs using the formulas proposed by Wan et al [36] and Luo et al [37].

### Risk of Bias Assessment

The methodological quality of the RCTs was assessed using the Cochrane Risk of Bias 2.0 tool (RoB 2.0) [38], which includes 5 domains and 1 overall judgment. The 5 domains are bias arising from the randomization process, bias due to deviations from intended interventions, bias due to missing outcome data, bias in the measurement of outcomes, and bias in the selection of the reported result. On the basis of responses to a series of signaling questions, each domain was judged as having “low risk of bias,” “some concerns,” or “high risk of bias.” Two independent reviewers (HW and YL) assessed the quality of each eligible study.

### Quality of Evidence Evaluation

The quality of evidence was further evaluated using the Grading of Recommendations Assessment, Development and Evaluation (GRADE) approach, which classifies evidence into 4 levels: high, moderate, low, and very low [39]. The certainty of evidence from RCTs was initially rated as high and was subsequently downgraded based on risk of bias, imprecision, inconsistency, indirectness, and publication bias [40-44].

### Data Synthesis and Analysis

All extracted data were independently entered by 2 reviewers and cross-checked by a third reviewer. For continuous outcomes measured using the same scale or scales that could be converted to a common metric, mean differences (MDs) with 95% CIs were calculated. Visual analog scale scores ranging from 0 to 100 were rescaled to a 0 to 10 metric and pooled with Numerical Rating Scale scores. If outcome scales could not be harmonized, standardized mean differences were used.

As clinical and methodological heterogeneity was expected across telerehabilitation modalities, supervision levels, intervention durations, and comparator intensities, all meta-analyses were conducted using random effects models rather than selecting fixed effect or random effects models according to statistical heterogeneity alone [45,46]. Random effects models were fitted using the restricted maximum likelihood estimator with the Hartung-Knapp-Sidik-Jonkman adjustment, which has been recommended as a more robust approach than conventional DerSimonian-Laird methods, particularly when the number of studies is small [47]. Statistical heterogeneity was assessed using the chi-square test, the *I*² statistic, and between-study variance (*τ*²). As *I*² quantifies relative heterogeneity but does not describe the expected distribution of true effects across different clinical settings, 95% prediction intervals (PIs) were calculated for the main random-effects meta-analyses where statistically estimable and clinically interpretable [48]. PIs were calculated using the confidence distribution approach proposed by Nagashima et al [49], which accounts for uncertainty in the between-study variance. PIs were not calculated for subgroup analyses because several subgroups included a limited number of studies, which would yield unstable and difficult-to-interpret intervals. In addition, previously reported minimal clinically important difference (MCID) thresholds were referenced to evaluate the clinical relevance of the estimated effects.

For multiarm trials, all potentially eligible arms were assessed against the predefined PICOS criteria. When multiple telerehabilitation arms were eligible for an outcome-specific meta-analysis, we selected the comparison most closely aligned with the prespecified PICOS framework and avoided double-counting participants or shared comparator groups.

Sensitivity analyses were performed using the leave-one-out method to evaluate the robustness of the pooled effect estimates. Additional sensitivity analyses were conducted by excluding studies with very small sample sizes, defined as fewer than 20 participants per group, and studies judged to be at high risk of bias. When 10 or more studies were available, small-study effects were assessed using funnel plots and Egger regression tests, which were interpreted as indicators of small-study effects rather than as definitive evidence of publication bias [50]. Analyses were implemented in R (meta package; R Foundation for Statistical Computing).

To explore potential sources of heterogeneity, subgroup analyses were conducted according to comparator intensity, delivery mode, and supervision level when at least two studies were available in each relevant subgroup. Comparator intensity was categorized as active control or minimal or nonactive control based on the actual treatment components, intensity of supervision, and degree of individualization received by the control group, as specified in the eligibility criteria. Delivery mode was categorized as self-directed or asynchronous or therapist-supported or synchronous. Subgroup analyses by supervision level used the operational low, moderate, and high categories described in the data extraction section. Analyses by delivery mode and supervision level were not specified in the registered protocol or subsequent protocol amendment and were therefore considered post hoc and exploratory. As subgroup comparisons were made across trials and were not protected by the randomization structure of the original RCTs, all subgroup findings were interpreted as observational and hypothesis generating rather than as causal evidence. Interpretation was further limited by the small number of studies in several subgroups and by the nonmutual independence of comparator intensity, delivery mode, and supervision level.

## Results

### Search Results and Study Selection

**Figure 1.**
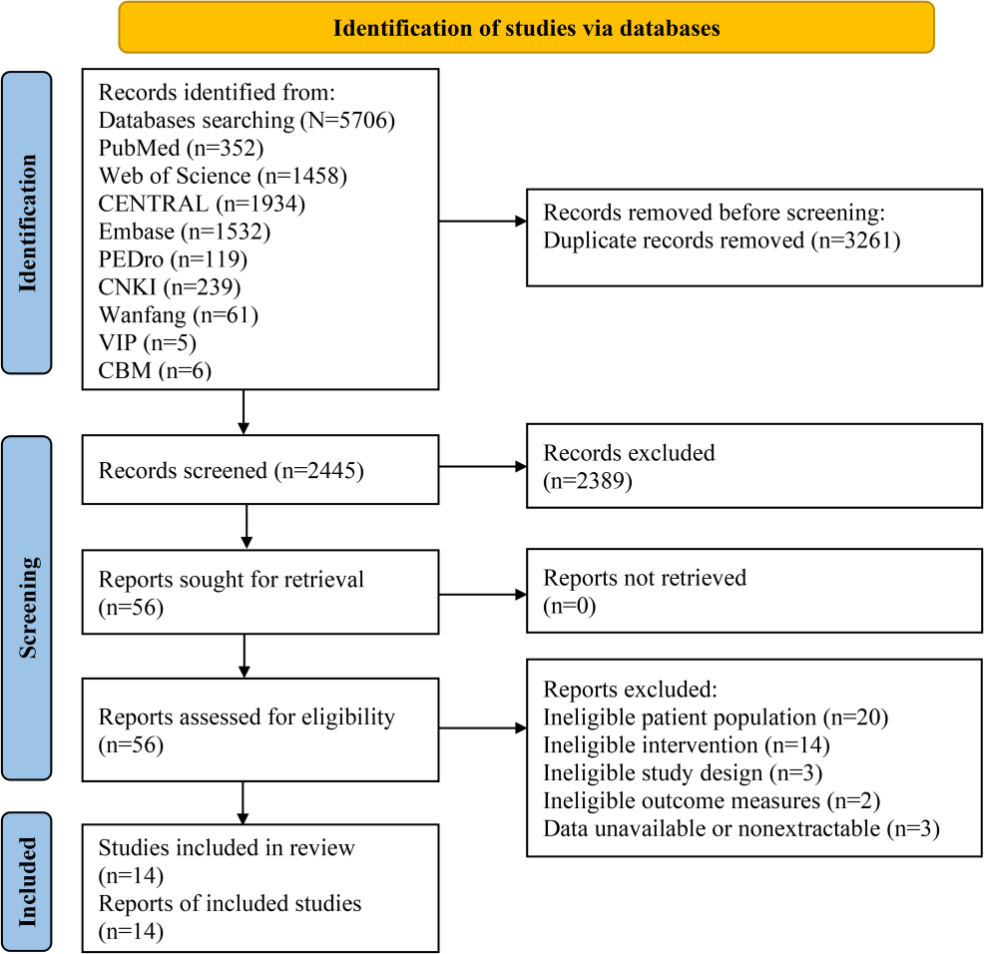
PRISMA (Preferred Reporting Items for Systematic Reviews and Meta-Analyses) flow diagram of study selection.

A total of 5706 records were initially identified through database searching, of which 3261 (57.1%) were removed as duplicates. Following title and abstract screening, 56 (2.3%) articles underwent full-text review. After full-text assessment, 42 (75%) studies were excluded for the following reasons: ineligible patient population (n=20, 47.6%), ineligible intervention (n=14, 33.3%), ineligible study design (n=3, 7.1%), ineligible outcome measures (n=2, 4.8%), and data unavailable or nonextractable (n=3, 7.1%). The full list of excluded full-text studies, with reasons for exclusion, is provided in Multimedia Appendix 3. Ultimately, 14 studies were included in this meta-analysis (Figure 1).

### Study Characteristics

These 14 studies involved a total of 794 patients diagnosed with CNSLBP. The main characteristics of the included studies, including sample size, sex distribution, interventions, and outcome measures, are summarized in Table 1. All included studies adopted an RCT design. Of these, 2 (14.3%) studies [51,52] used a 3-arm parallel-group design, whereas the remaining 12 (85.7%) studies [53-64] used a 2-arm parallel-group design. Stratified by comparator intensity, 6 (42.9%) studies [51,54,55,59,62,63] were classified as active control trials, and 8 (57.1%) studies [52,53,56-58,60,61,64] were classified as minimal or nonactive control trials.

**Table 1.** Characteristics of included randomized controlled trials.

Study (y), country, and group	Sample size	Gender, n (%)		Age (y), mean (SD)	Intervention	Duration	Outcomes
		Male	Female				
Almhdawi et al (2020) [53], Jordan							VAS^a^, ODI^b^
Exp^c^	21	7 (33.3)	14 (66.7)	40.48 (7.22)	Smartphone app (“Relieve My Back”) providing education, office-based stretching exercises, and home-based strengthening exercises	Daily for 6 wk	
Con^d^	20	12 (60)	8 (40)	41.70 (6.53)	Placebo smartphone app providing general nutrition information only	Daily for 6 wk	
Dadarkhah et al (2021) [54], Iran							VAS, ODI
Exp	28	16 (57.1)	12 (42.9)	49 (9.3)	Home-based training with telephone follow-up	Twice daily for 4 wk	
Con	28	16 (57.1)	12 (42.9)	50 (8.6)	Face-to-face in-clinic training	Three times weekly for 4 wk	
Fatoye et al (2020) [55], Nigeria							ODI
Exp	21	—^e^	—	47.3 (11.6)	McKenzie therapy delivered via a mobile app	Three times weekly for 8 wk	
Con	26	—	—	50 (10.7)	Hospital-based McKenzie therapy	Three times weekly for 8 wk	
Feng et al (2025) [56], China							NRS^f^
Exp	39	22 (56.4)	17 (43.6)	27.33 (9.63)	mHealth^g^ app providing patient education, health coaching, and structured exercise programs	Three times weekly for 8 wk	
Con	39	17 (43.6)	22 (56.4)	26.67 (11.85)	Standard care, including patient education and printed home exercise instructions	Three times weekly for 8 wk	
Groenveld et al (2023) [57], The Netherlands							ODI
Exp	20	3 (15)	17 (85)	51 (2.9)	Self-administered behavioral therapy–based virtual reality application	10 min daily for 4 wk (≤30 min per session and ≤3 sessions per day)	
Con	20	4 (20)	16 (80)	52 (2.5)	Standard care	—	
Karaduman and Ataş Balci (2024) [51], Turkey							NRS, ODI, TSK^h^
Exp 1	22	9 (40.9)	13 (59.1)	46.31 (12.34)	Physiotherapist-guided stabilization exercises via videoconferencing	20‐30 min, 3 times weekly for 4 wk	
Exp 2	22	7 (31.8)	15 (68.2)	43.95 (17.06)	Handout-guided home stabilization exercises with telephone support during weeks 1‐3	20‐30 min, 3 times weekly for 4 wk	
Con	22	20 (90.9)	2 (9.1)	46.50 (12.73)	Hospital-based physiotherapist-supervised stabilization exercises	20‐30 min, 3 times weekly for 4 wk	
Lara-Palomo et al (2022) [58], Spain							VAS, ODI, TSK
Exp	39	17 (43.6)	22 (56.4)	41.9 (9.4)	Online McKenzie therapy plus TENS^i^	Three times weekly for 8 wk	
Con	35	14 (40)	21 (60)	54.6 (12.9)	Self-administered home-based McKenzie therapy plus TENS	Three times weekly for 8 wk	
López-Marcos et al (2024) [59], Spain							NRS, ODI
Exp	45	—	—	49.9 (10)	Mobile health app–based exercise program without in-person supervision	Three times weekly for 12 wk	
Con	45	—	—	52.2 (9.8)	Same mobile health app–based exercise program with in-person supervised training	Three times weekly for 12 wk	
Özden et al (2022) [60], Turkey							VAS, ODI, TSK
Exp	25	11 (44)	14 (56)	40.1 (1.6)	Video exercise–based telerehabilitation software	8 wk	
Con	25	9 (36)	16 (64)	42.3 (1.6)	Home exercises guided by paper-based instructions	8 wk	
Özden et al (2024) [61], Turkey							VAS, ODI
Exp	22	—	—	43.5 (12.6)	Video exercise–based home exercise program with visual feedback	Daily (2×10 repetitions) for 8 wk	
Con	22	—	—	46.2 (12.4)	Same video exercise program without visual feedback	Daily (2×10 repetitions) for 8 wk	
Tawfek and Çil (2025) [52], Turkey							VAS, ODI, TSK
Exp 1	24	15 (62.5)	9 (37.5)	43.21 (12.53)	Real-time therapist-guided telerehabilitation delivered via a dedicated app	Twice weekly for 12 wk	
Exp 2	24	12 (50)	12 (50)	38.79 (10.39)	Self-administered exercise program using prerecorded videos with weekly email follow-up	12 wk	
Con	24	14 (58.3)	10 (41.7)	41.79 (10.01)	Unsupervised home exercise program	12 wk	
Villatoro-Luque et al (2023) [62], Spain							VAS, TSK
Exp	34	19 (55.9)	15 (44.1)	41.85 (10.37)	App-based exercise program plus pain neuroscience education	Twice weekly for 8 wk	
Con	34	15 (44.1)	19 (55.9)	44.29 (11.19)	In-clinic supervised exercise program with identical educational content	Twice weekly for 8 wk	
Yang et al (2019) [63], China							VAS
Exp	5	4 (80)	1 (20)	35 (10.93)	In-clinic physical therapy plus a smartphone app–based self-management program	4 wk	
Con	3	0 (0)	3 (100)	50.33 (9.29)	Routine in-clinic physical therapy alone	4 wk	
Zadro et al (2019) [64], Australia							NRS, TSK
Exp	30	12 (40)	18 (60)	68.8 (5.5)	Unsupervised home-based exercise program using software and a gaming console	60 min, 3 times weekly for 8 wk	
Con	30	17 (56.7)	13 (43.3)	67.8 (6)	No specific intervention; participants maintained their usual daily activities	—	

a VAS: visual analog scale.

b ODI: Oswestry Disability Index.

c Exp: experimental group.

d Con: control group.

e Not available.

f NRS: Numerical Rating Scale.

g mHealth: mobile health.

h TSK: Tampa Scale of Kinesiophobia.

i TENS: transcutaneous electrical nerve stimulation.

### Intervention Characteristics and Comparator Classification

The included trials evaluated diverse telerehabilitation models, including app-based programs, web- or video-based exercise, virtual reality, telephone-supported exercise instruction, and synchronous videoconference-based telerehabilitation. Delivery mode, contact format, exercise prescription and progression, therapist supervision, feedback mechanisms, adherence monitoring, safety or symptom exacerbation monitoring, and comparator intensity varied substantially across studies. Comparator conditions were classified as active controls or minimal or nonactive controls based on the actual treatment components, supervision intensity, and degree of individualization. Detailed intervention characteristics, rehabilitation process features, comparator classifications, and classification rationales are presented in Table 2.

**Table 2.** Intervention characteristics and comparator classification.

Study	Telerehabilitation modality	Delivery mode and contact format	Exercise prescription or progression	Therapist supervision and feedback^a^	Adherence and safety monitoring	Comparator description	Comparator classification	Classification rationale
Almhdawi et al, 2020 [53]	Smartphone app–based program	Asynchronous, self-directed digital delivery	LBP^b^ advice or instructions, office-based stretching, home-based strengthening for lower back and abdominal muscles, posture reminders, and walking break reminders; progression not reported.	Low: automated app reminders only; no individualized therapist feedback or real-time supervision reported.	Google Firebase logs recorded app use. Adverse event or symptom exacerbation monitoring was not reported.	Placebo smartphone app providing general nutrition advice and nutrition-related notifications; participants were allowed to continue traditional medical care.	Minimal or nonactive control	Classified as minimal or nonactive because the comparator did not provide LBP-specific rehabilitation, therapeutic exercise, or therapist-supervised care; it functioned as a placebo digital control with non-LBP nutritional information
Dadarkhah et al, 2021 [54]	Telephone-supported remote exercise instruction	Home-based remote delivery with scheduled telephone follow-up	Structured core stability, flexibility, strengthening, warm-up, and cool-down exercises; written illustrated exercise guide and workout logbook; progression not clearly reported.	Moderate: physical therapist telephone calls of approximately 10 min, 3 d/wk for 4 wk; calls assessed barriers, pain increase, adverse events, and encouraged completion.	Workout logbook and telephone follow-up were used to monitor exercise completion, barriers, self-efficacy, pain increase, and adverse events.	In-person exercise instruction at the clinic using the same exercise program, delivered 3 times per week for 4 wk.	Active control	Classified as active because the comparator received structured clinic-based exercise instruction, equivalent in therapeutic content and delivered as in-person rehabilitation
Fatoye et al, 2020 [55]	Mobile phone app–based McKenzie therapy	Home-based mobile app–assisted delivery	McKenzie extension protocol, including extension lying prone, extension in prone, extension in standing, plus back care education; progress tracking reported but progression rules not clearly described.	Moderate: performance feedback and progress tracking were telemonitored through caregiver support; SMS text messages and reminder calls were provided 3 times per week; no real-time therapist supervision reported.	Performance feedback and progress tracking were telemonitored through caregiver support; SMS text messages and reminder calls supported adherence. Adverse event or symptom exacerbation monitoring was not clearly reported.	Clinic-based McKenzie therapy, including the same McKenzie extension protocol and back care education, delivered in outpatient physiotherapy or clinic setting	Active control	Classified as active because the comparator received structured clinic-based physiotherapy using the McKenzie extension protocol and back care education, rather than minimal care or advice only
Feng et al, 2025 [56]	mHealth^c^ app–supported structured exercise with WeChat video health coaching	Mixed app-based asynchronous exercise plus weekly synchronous group video coaching	Individualized app-based exercise prescription based on FITT-VP^d^ principles; phased goals were set through the app; treatment plan could be advanced during weekly video coaching.	Moderate: weekly WeChat group video coaching with a physical therapist; movement accuracy was checked, and the treatment plan could be advanced if goals were met; not every exercise session was supervised in real time.	App reminders and goal tracking supported adherence. Attendance, medication changes, and adverse events were recorded during follow-up.	Paper-based home exercise manual with the same training movements and frequency; app-based patient education; initial therapist instruction only, with no routine follow-up instruction unless patients requested help.	Minimal or nonactive control	Classified as minimal or nonactive because the comparator mainly consisted of paper-based unsupervised home exercise and education after initial instruction, without scheduled therapist supervision, routine feedback, or structured individualized follow-up during the 8-wk treatment period
Groenveld et al, 2023 [57]	Behavioral therapy–based VR^e^ application	Self-administered home-based VR delivery	VR-based pain education and behavioral therapy exercises incorporating acceptance and commitment therapy, mindfulness, hypnotherapy, and eye movement desensitization and reprocessing-related principles; fixed self-administered program, not primarily exercise based.	Low: initial setup and research-staff telephone calls were used for adherence, adverse event, and technical monitoring only; no scheduled individualized therapist-led rehabilitation feedback or real-time supervision was reported.	Telephone calls on days 7 and 14 monitored adherence, adverse events, and technical problems; mild dizziness was recorded as an adverse event.	Standard care while waiting for follow-up pain clinic visit; no additional VR intervention or structured rehabilitation provided.	Minimal or nonactive control	Classified as minimal or nonactive because the comparator received standard care or waiting list care without additional structured rehabilitation, therapist-supervised exercise, or individualized active treatment during the 4-wk intervention period
Karaduman and Ataş Balci, 2024 [51]	Video conference–based tele-supervised stabilization exercise	Synchronous tele-supervised exercise sessions	Standardized stabilization exercise protocol, including abdominal bracing and progressive exercises in supine, crawling, and standing positions; progression based on ability to sustain abdominal bracing and complete repetitions.	High: real-time physiotherapist guidance via videoconference during exercise sessions	Adherence and adverse event or symptom exacerbation monitoring were not clearly reported.	In-person-supervised stabilization exercises delivered at the hospital under physiotherapist guidance.	Active control	Classified as active because the comparator received the same structured stabilization exercise protocol under in-person physiotherapist supervision in a hospital setting
Lara-Palomo et al, 2022 [58]	Internet-based eHealth rehabilitation program	Asynchronous web or mobile-based delivery	Individualized McKenzie exercises and TENS^f^ or electroanalgesia based on McKenzie classification; progression not clearly reported.	Low: online platform provided video or audio instructions and recorded adherence; no real-time therapist supervision or scheduled individualized feedback reported.	The online platform-recorded adherence through logins and time spent. Adverse event or symptom exacerbation monitoring was not reported.	Home rehabilitation program with McKenzie exercises and TENS, taught during 2 initial sessions, then performed at home using printed instructions or booklet	Minimal or nonactive control	Classified as minimal or nonactive because the comparator was an unsupervised home rehabilitation program after initial instruction, without ongoing therapist supervision or online feedback
López-Marcos et al, 2024 [59]	mHealth app–based therapeutic exercise self-management	App-based self-management exercise program	Therapeutic exercise program based on adapted McGill Big Three exercises, including warm-up, motor control, and strengthening exercises; patients selected levels across predefined basic, intermediate, advanced, and expert programs.	Moderate: app-based self-management with access to individualized remote consultation or chat support and app-based adherence or outcome monitoring; no real-time therapist supervision of each session was provided.	The app recorded exercise adherence and patient-reported outcomes; safety or symptom exacerbation monitoring was not clearly reported.	Same mHealth app–based self-management program plus scheduled face-to-face supervision sessions.	Active control	Classified as active because the comparator received the same exercise-based self-management program with additional scheduled face-to-face physiotherapist supervision, representing a higher-intensity active rehabilitation comparator
Özden et al, 2022 [60]	Video exercise–based telerehabilitation software	Asynchronous web-based home exercise delivery with clinician communication	Home exercise program, including stretching, strengthening, bridging, spine mobility, McKenzie extension, and Williams flexion exercises; minor patient-specific modifications or progressions could be provided.	Moderate: Fizyoweb monitored online activity and presented statistical data to the clinician; messaging platform allowed patient-clinician communication; no real-time supervision reported.	Fizyoweb monitored online activity and provided statistical data to the clinician. Adverse event or symptom exacerbation monitoring was not clearly reported.	Paper-based conventional home exercise program with the same exercises provided as printed instruction forms	Minimal or nonactive control	Classified as minimal or nonactive because the comparator consisted of paper-based home exercises without digital monitoring, clinician communication through the software, or structured supervision
Özden et al, 2024 [61]	Visual feedback–based clinical monitoring application	App-based video exercise program with PROM^g^-based teleassessment and graphical feedback	Video exercise–based home program, including stretching, strengthening, core stabilization, Williams flexion, and McKenzie extension exercises; exercise protocols were determined according to patient needs.	Moderate: PhysioAnalyst collected PROMs and provided graph or table-based visual feedback; physiotherapists could observe patient progress through the same graphs; no live session supervision reported.	PROMs and exercise adherence were assessed through PhysioAnalyst/EARS^h^. Safety or symptom exacerbation monitoring was not clearly reported	Same video exercise protocol delivered without graph-based visual feedback after the week 4 assessment	Minimal or nonactive control	Classified as minimal or nonactive because the comparator received the same home video exercise protocol but without the additional visual feedback, self-monitoring, or feedback-supported monitoring component
Tawfek and Çil, 2025 [52]	Synchronous exercise-based telerehabilitation	Real-time videoconference-based telerehabilitation	Structured exercise program, including warm-up, core stabilization, stretching, and cool-down exercises, combined with patient education or pain neuroscience education	High: real-time therapist-led Zoom sessions twice weekly for 12 wk, with live demonstration and feedback	Session completion was followed. Detailed adverse event or symptom exacerbation monitoring was not clearly reported.	Unsupervised home-based exercise program delivered through a digital book	Minimal or nonactive control	Classified as minimal or nonactive because the comparator received an unsupervised home exercise program without real-time therapist supervision or structured interactive feedback
Villatoro-Luque et al, 2023 [62]	Mobile app or video-based telerehabilitation with videoconference follow-up	Home-based telerehabilitation with instructional videos and weekly videoconferencing	Exercise-based telerehabilitation program plus pain neurophysiology education; same exercises as clinic group; exercise intensity was guided by pain response.	Moderate: weekly videoconferencing follow-up with physiotherapist; patients could contact the physiotherapist for exercise-related problems; not every exercise session was supervised in real time.	Pain during exercise was monitored during follow-up. Adverse event or symptom exacerbation monitoring was not clearly reported.	Same pain education and exercise program delivered in a clinic facility under clinician supervision	Active control	Classified as active because the comparator received the same structured exercise and pain education program in a clinic setting under clinician supervision
Yang et al, 2019 [63]	Smartphone app–based self-management plus physiotherapy	App-supported self-management combined with physiotherapy	Individualized exercises prescribed by therapist; therapist could modify exercises according to feedback and symptoms; app reminders supported exercise performance.	Moderate: Pain Care app reminders 4 times daily; patients recorded pain and activity; therapist could adjust exercises based on feedback and symptoms.	Pain intensity and activity levels were recorded through the app. Safety or adverse event monitoring was not reported.	Physiotherapy only, which could include manual therapy, electrophysical therapy, and traction as prescribed by the physiotherapist.	Active control	Classified as active because the comparator received physiotherapy delivered or prescribed by a physiotherapist rather than no treatment, education only, or unsupervised self-care
Zadro et al, 2019 [64]	Home-based video game exercise program	Self-directed Nintendo Wii Fit U–based home exercise	Wii Fit U flexibility, strengthening, and aerobic exercises; initial physiotherapist screening removed unsafe or pain-provoking exercises; progression focused on increasing repetitions or selecting more challenging exercises.	Moderate: initial home visit by physiotherapist to set up equipment and guide the first session; Wii Fit U provided video or instructions and performance feedback; fortnightly telephone calls supported progression.	Fortnightly telephone calls monitored progression, adverse events, and equipment issues; no adverse events were reported.	Usual activities, including usual care–seeking behaviors	Minimal or nonactive control	Classified as minimal or nonactive because the comparator was instructed to continue usual activities without a structured rehabilitation program or therapist-supervised exercise.

a Therapist supervision was categorized as low, moderate, or high according to the degree of therapist involvement, scheduled contact, monitoring, and individualized feedback. Low supervision indicated self-directed or automated interventions without scheduled individualized therapist contact or feedback; moderate supervision indicated scheduled asynchronous or periodic therapist contact, remote monitoring, adherence support, or individualized non–real-time feedback; and high supervision indicated synchronous therapist-led sessions with real-time guidance, exercise correction, and individualized feedback.

b LBP: low back pain.

c mHealth: mobile health.

d FITT-VP: frequency, intensity, time, type, volume, and progression.

e VR: virtual reality.

f TENS: transcutaneous electrical nerve stimulation.

g PROM: patient-reported outcome measure.

h EARS: Exercise Adherence Rating Scale.

### Risk of Bias

**Figure 2.**
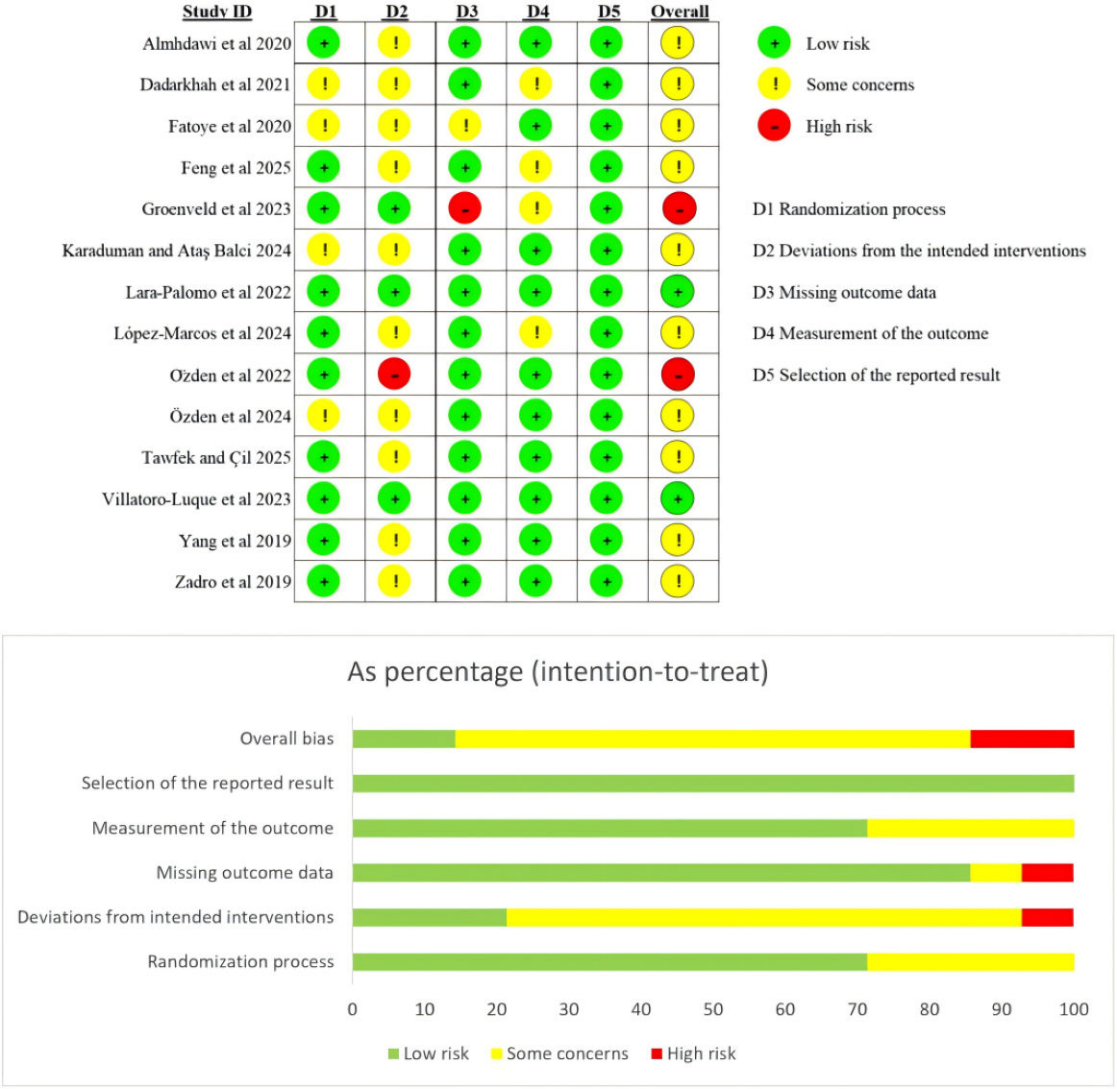
Risk of bias assessment using RoB 2.0 [51-64].

Risk of bias was assessed using the Cochrane RoB 2.0 tool (Figure 2). Overall, 10 (71.4%) studies [51-56,59,61,63,64] were judged to have some concerns, 2 (14.3%) studies [57,60] were rated as being at high risk of bias, and 2 (14.3%) studies [58,62] were judged to be at low risk of bias. The main sources of bias were related to the randomization process, deviations from intended interventions, and outcome measurement. Several trials provided insufficient information on sequence generation, allocation concealment, or baseline comparability. In addition, blinding of participants and therapists was generally difficult because of the nature of telerehabilitation and exercise-based interventions. As the main outcomes were patient-reported, including pain intensity, functional disability, and kinesiophobia, participants’ awareness of group allocation may have influenced adherence, expectations, or outcome reporting. The 2 studies rated as high risk of bias were mainly affected by missing outcome data and deviations from intended interventions.

### Outcomes

#### Pain Intensity

**Figure 3.**
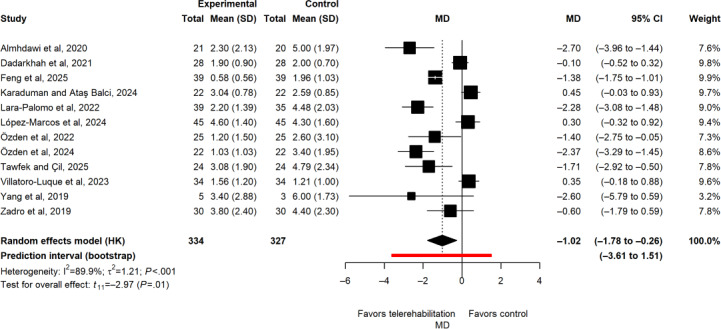
Forest plot of pain intensity [51-54,56,58-64]. MD: mean difference.

A total of 12 (85.7%) studies were included in the meta-analysis of pain intensity (Figure 3). The random-effects meta-analysis showed that telerehabilitation was associated with a statistically significant average reduction in pain intensity compared with control conditions (MD −1.02, 95% CI −1.78 to −0.26; *P*=.01). However, heterogeneity was substantial (*I*²=89.9%; *τ*²=1.21). The 95% PI crossed the null (95% PI −3.61 to 1.51), indicating that the effect may vary considerably across clinical settings and intervention models. Therefore, although the average effect favored telerehabilitation, the consistency and generalizability of this effect remain uncertain. The certainty of evidence for this outcome was rated as low, due mainly to risk of bias and inconsistency.

**Figure 4.**
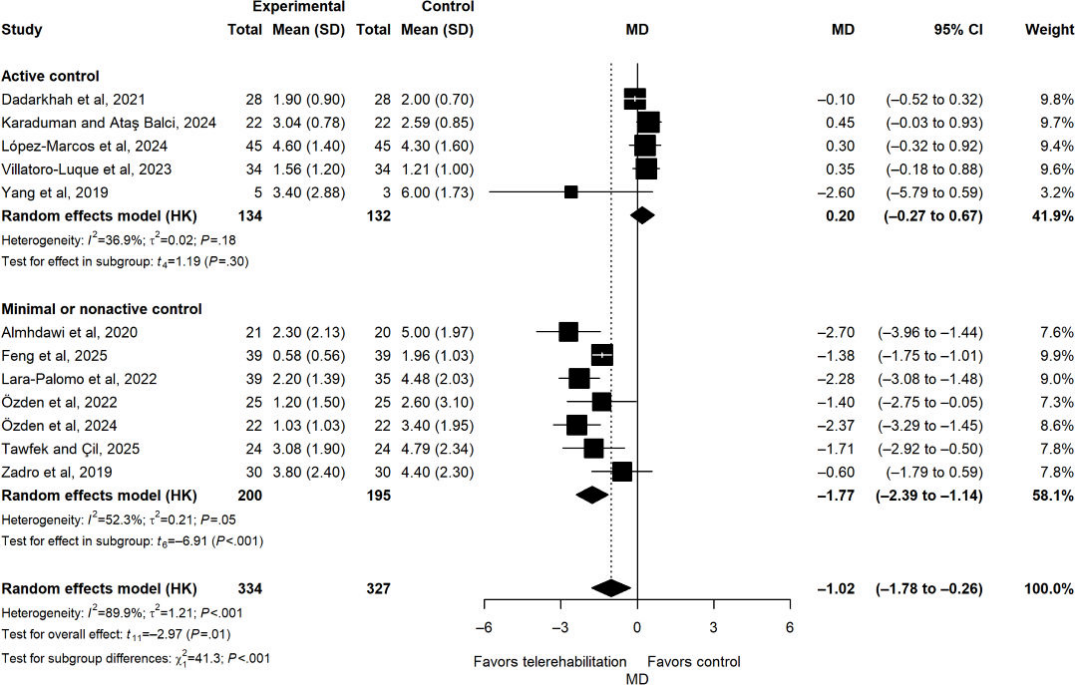
Subgroup analysis of pain intensity by comparator intensity [51-54,56,58-64]. MD: mean difference.

Subgroup analysis by comparator intensity showed no statistically significant difference between telerehabilitation and active controls (MD 0.20, 95% CI −0.27 to 0.67; *P*=.30). In contrast, telerehabilitation was associated with greater pain reduction compared with minimal or nonactive controls (MD −1.77, 95% CI −2.39 to −1.14; *P*<.001). The test for subgroup differences was statistically significant (*Q*_b_=41.27; *P*<.001), suggesting that comparator intensity may have contributed to the observed heterogeneity (Figure 4). However, this finding should still be interpreted cautiously given the low or very low certainty of the subgroup estimates.

Subgroup analysis by delivery mode showed that self-directed or asynchronous interventions were associated with a statistically significant reduction in pain intensity (MD −1.93, 95% CI −2.95 to −0.90; *P*=.01), whereas therapist-supported or synchronous interventions showed no statistically significant effect (MD −0.38, 95% CI −1.32 to 0.55; *P*=.35; Figure S1 in Multimedia Appendix 4). The subgroup difference was statistically significant (Qb=8.46; *P*=.004). Subgroup analysis by supervision level also showed a significant subgroup difference for pain intensity (Q_b_=16.81; *P*<.001; Figure S2 in Multimedia Appendix 4), although this finding should be interpreted cautiously because the low- and high-supervision subgroups each included only 2 studies and the high-supervision estimate was highly imprecise. As comparator intensity, delivery mode, and supervision level were not mutually independent, these subgroup findings should not be interpreted as definitive evidence that one telerehabilitation model was superior to another.

#### Functional Disability

**Figure 5.**
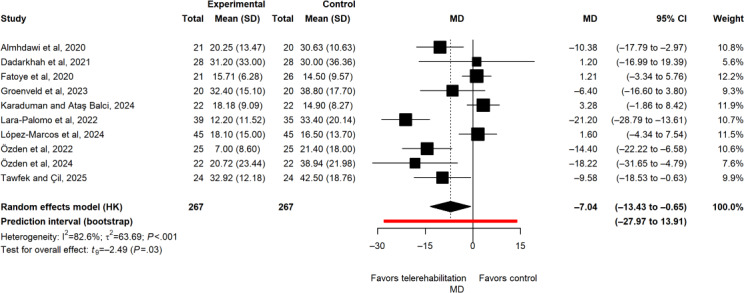
Forest plot of functional disability [51-55,57-61]. MD: mean difference.

A total of 10 (71.4%) studies were included in the meta-analysis of functional disability (Figure 5). The random-effects meta-analysis showed that telerehabilitation was associated with a statistically significant average reduction in functional disability compared with control conditions (MD −7.04, 95% CI −13.43 to −0.65; *P*=.03). However, heterogeneity was substantial (*I*²=82.6%; *τ*²=63.70). The 95% PI crossed the null (95% PI −27.97 to 13.91), indicating that the effect may vary considerably across clinical settings and intervention models. Therefore, although the average effect favored telerehabilitation, the consistency and generalizability of this effect remain uncertain. The certainty of evidence for this outcome was rated as low, mainly because of risk of bias and inconsistency.

**Figure 6.**
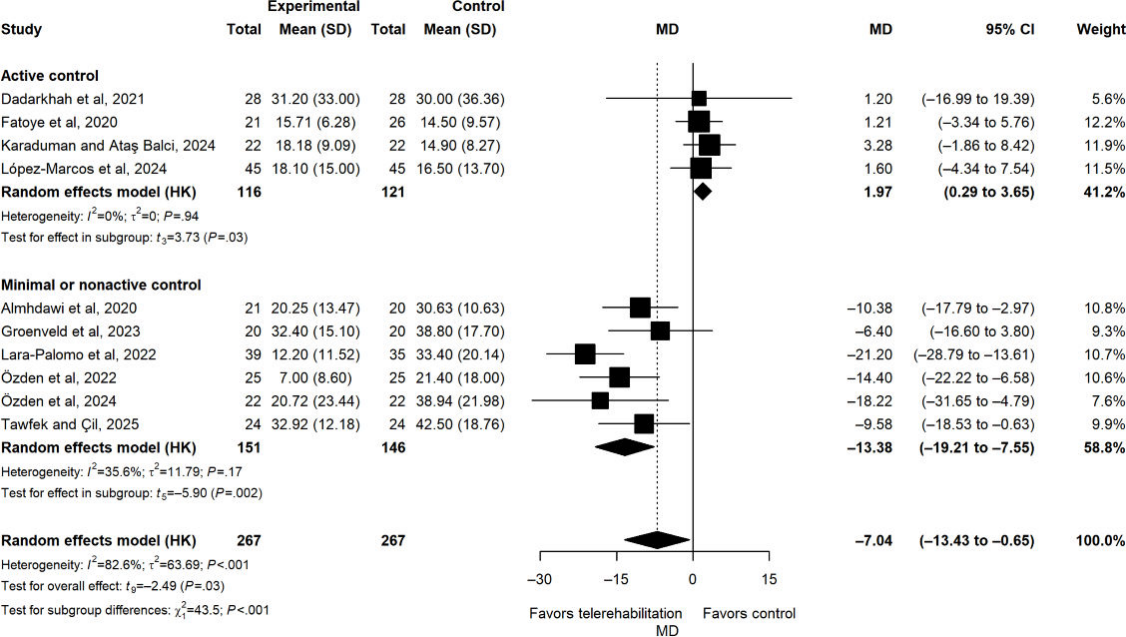
Subgroup analysis of functional disability by comparator intensity [51-55,57-61]. MD: mean difference.

Subgroup analysis by comparator intensity showed that telerehabilitation was associated with a small but statistically significant disadvantage compared with active controls (MD 1.97, 95% CI 0.29 to 3.65; *P*=.03). As higher Oswestry Disability Index scores indicate greater disability, this result favored active control conditions. In contrast, telerehabilitation was associated with greater improvement compared with minimal or nonactive controls (MD −13.38, 95% CI −19.21 to −7.55; *P*=.002). The test for subgroup differences was statistically significant (*Q*_*b*_=43.47; *P*<.001), suggesting that comparator intensity may have contributed to the observed heterogeneity (Figure 6). This pattern should be interpreted cautiously because the subgroup estimates were based on low-certainty evidence.

Subgroup analysis by delivery mode showed that self-directed or asynchronous interventions were associated with greater improvement in functional disability (MD −14.12, 95% CI −21.40 to −6.84; *P*=.006), whereas therapist-supported or synchronous interventions showed no statistically significant effect (MD 0.57, 95% CI −4.82 to 5.96; *P*=.79; Figure S3 in Multimedia Appendix 4). The subgroup difference was statistically significant (*Q*_*b*_=20.28; *P*<.001). However, because delivery mode was not independent of comparator intensity and supervision level, this exploratory finding should not be interpreted as evidence that self-directed or asynchronous interventions are superior to therapist-supported or synchronous interventions. Exploratory subgroup analysis by supervision level did not show a statistically significant subgroup difference for functional disability (*Q*_b_=2.42; *P*=.30; Figure S4 in Multimedia Appendix 4).

#### Kinesiophobia

**Figure 7.**
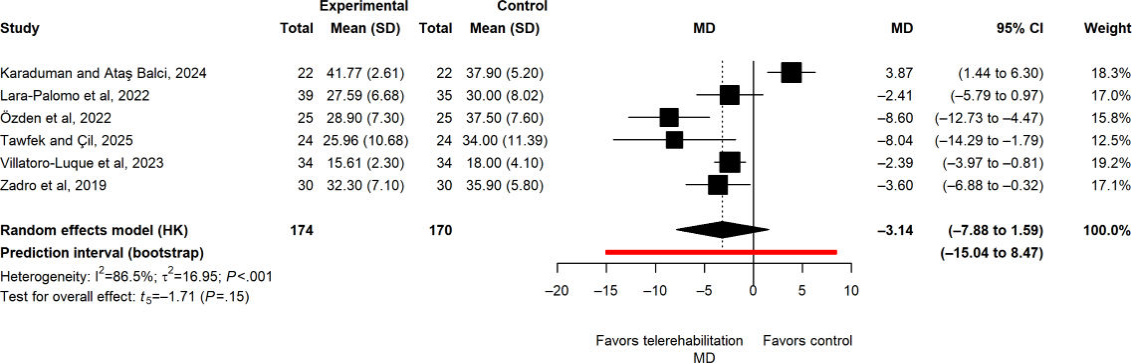
Forest plot of kinesiophobia [51,52,58,60,62,64]. MD: mean difference.

A total of 6 (42.9%) studies were included in the meta-analysis of kinesiophobia (Figure 7). The random-effects meta-analysis showed that telerehabilitation was not associated with a statistically significant average reduction in kinesiophobia compared with control conditions (MD −3.14, 95% CI −7.88 to 1.59; *P*=.15). Heterogeneity was substantial (*I*²=86.5%; τ²=16.95). The 95% PI crossed the null (95% PI −15.04 to 8.47), indicating substantial uncertainty in the expected effect across clinical settings and intervention models. Therefore, the evidence does not support a reliable average effect or a consistent benefit of telerehabilitation for kinesiophobia. The certainty of evidence for this outcome was rated as very low, mainly because of risk of bias, inconsistency, and imprecision.

**Figure 8.**
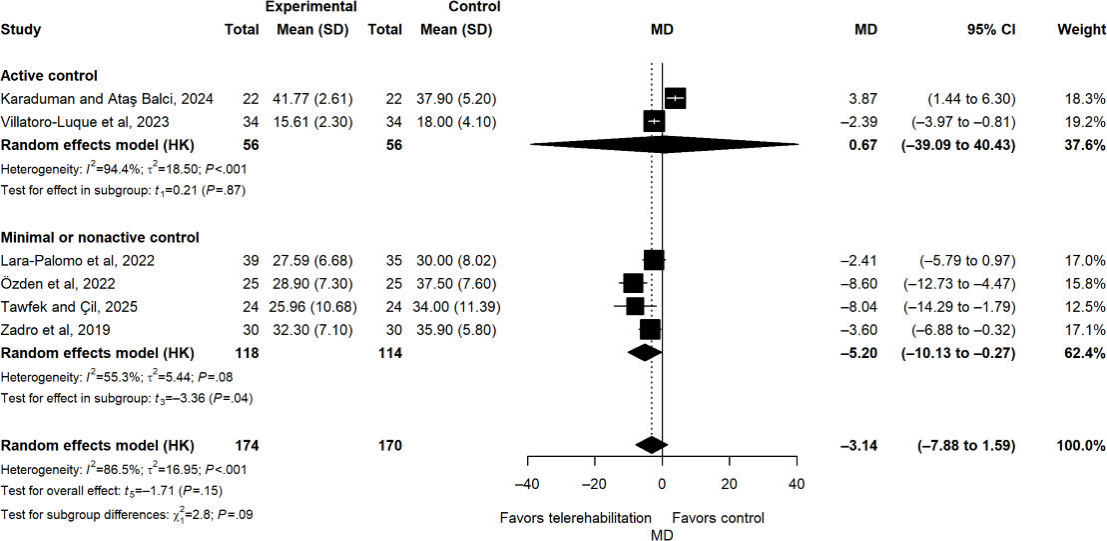
Subgroup analysis of kinesiophobia by comparator intensity [51,52,58,60,62,64]. MD: mean difference.

Subgroup analysis by comparator intensity showed no statistically significant difference between telerehabilitation and active controls for kinesiophobia (MD 0.67, 95% CI −39.09 to 40.43; *P*=.87). In studies using minimal or nonactive controls, telerehabilitation was associated with lower kinesiophobia scores (MD −5.20, 95% CI −10.13 to −0.27; *P*=.04). However, the test for subgroup differences was not statistically significant (*Q*_*b*_=2.83; *P*=.09), and the estimate for active controls was highly imprecise (Figure 8). Given the very low certainty of the subgroup estimates, these findings should be interpreted cautiously and do not provide reliable evidence that comparator intensity modified the effect of telerehabilitation on kinesiophobia.

In the subgroup analysis by delivery mode, neither self-directed nor asynchronous interventions (MD −4.69, 95% CI −12.62 to 3.25; *P*=.13) nor therapist-supported or synchronous interventions (MD −1.72, 95% CI −16.11 to 12.66; *P*=.66; Figure S5 in Multimedia Appendix 4) showed a statistically significant effect on kinesiophobia. The subgroup difference was not statistically significant (*Q*_*b*_=0.60; *P*=.44). Subgroup analysis by supervision level was not formally summarized for kinesiophobia because fewer than 2 studies were available in one relevant subgroup.

### Quality of Evidence

The GRADE assessment is presented in Table 3. The certainty of evidence for most outcomes was low or very low, mainly due to risk of bias, inconsistency, and imprecision. In particular, blinding of the interventions was inherently difficult, and most outcomes were based on subjective reporting, which increased the risk of bias in some studies. Furthermore, the included studies were generally limited by small sample sizes, and variations in intervention modalities, comparator intensity, and follow-up schedules across studies further lowered the certainty of the evidence.

**Table 3.** Summary of GRADE^a^ certainty of evidence for telerehabilitation versus control conditions overall and by comparator type (minimal or nonactive control and active control) in adults with chronic nonspecific low back pain (setting: hospitals and homes).

Outcome	Studies (n)	Study design	Certainty assessment					Patients (n)		Relative effect (95% CI)	Absolute effect (95% CI)	Certainty	Importance
			Risk of bias	Inconsistency	Indirectness	Imprecision	Other considerations	Telerehabilitation	Comparator				
Overall comparison													
Pain	12	Randomized trials	Serious^b^	Serious^c^	Not serious	Not serious	None	334	327	—^d^	MD^e^ 1.02 lower (1.78 lower to 0.26 lower)	⨁⨁◯◯ Low^b^^,^^c^	Critical
Functional disability	10	Randomized trials	Serious^b^	Serious^c^	Not serious	Not serious	None	267	267	—	MD 7.04 lower (13.43 lower to 0.65 lower)	⨁⨁◯◯ Low^b^^,^^c^	Critical
Kinesiophobia	6	Randomized trials	Serious^b^	Serious^c^	Not serious	Serious^f^	None	174	170	—	MD 3.14 lower (7.88 lower to 1.59 higher)	⨁◯◯◯ Very low^b^^,^^c^^,^^f^	Critical
Minimal or nonactive comparator													
Pain	7	Randomized trials	Serious^b^	Serious^c^	Not serious	Serious^f^	None	200	195	—	MD 1.77 lower (2.39 lower to 1.14 lower)	⨁◯◯◯ Very low^b^^,^^c^^,^^f^	Critical
Functional disability	6	Randomized trials	Serious^b^	Not serious	Not serious	Serious^f^	None	151	146	—	MD 13.38 lower (19.21 lower to 7.55 lower)	⨁⨁◯◯ Low^b^^,^^f^	Critical
Kinesiophobia	4	Randomized trials	Serious^b^	Serious^c^	Not serious	Serious^f^	None	118	114	—	MD 5.20 lower (10.13 lower to 0.27 lower)	⨁◯◯◯ Very low^b^^,^^c^^,^^f^	Critical
Active comparator													
Pain	5	Randomized trials	Serious^b^	Not serious	Not serious	Serious^f^	None	134	132	—	MD 0.20 higher (0.27 lower to 0.67 higher)	⨁⨁◯◯ Low^b^^,^^f^	Critical
Functional disability	4	Randomized trials	Serious^b^	Not serious	Not serious	Serious^f^	None	116	121	—	MD 1.97 higher (0.29 higher to 3.65 higher)	⨁⨁◯◯ Low^b^^,^^f^	Critical
Kinesiophobia	2	Randomized trials	Serious^b^	Serious^c^	Not serious	Very serious^g^	None	56	56	—	MD 0.67 higher (39.09 lower to 40.43 higher)	⨁◯◯◯ Very low^b^^,^^c^^,^^g^	Critical

a GRADE: Grading of Recommendations Assessment, Development and Evaluation.

b Downgraded for risk of bias because most included trials were judged as having “some concerns” using the RoB 2 tool.

c Downgraded for inconsistency when substantial heterogeneity was present in the meta-analysis (*I*²>50%).

d Not applicable.

e MD: mean difference.

f Downgraded for imprecision when the pooled sample size was <400.

g Downgraded 2 levels for imprecision when the 95% CI was extremely wide and crossed both appreciable benefit and harm.

### Sensitivity Analysis

For pain intensity, the leave-one-out analysis showed that sequential omission of individual studies did not materially change the direction or statistical significance of the pooled effect, with pooled MDs ranging from −0.88 to −1.17 (Figure S1 in Multimedia Appendix 5). After excluding a study with fewer than 20 participants in either arm [63], the pooled effect remained statistically significant (MD −0.97, 95% CI −1.76 to −0.18; Figure S2 in Multimedia Appendix 5). Similarly, after excluding a study judged as having a high risk of bias [60], the pooled effect remained statistically significant (MD −1.00, 95% CI −1.83 to −0.17; Figure S3 in Multimedia Appendix 5). These findings suggest that the pain outcome was generally robust, although substantial heterogeneity persisted across sensitivity analyses.

For functional disability, the leave-one-out analysis showed that the direction of the pooled effect remained consistent, but statistical significance was not fully stable. Sequential omission of individual studies yielded pooled MDs ranging from −5.15 to −8.41, and the 95% CI crossed the null after removal of several studies (Figure S4 in Multimedia Appendix 5). After excluding studies at high risk of bias [57,60], the pooled effect was attenuated and no longer statistically significant (MD −6.21, 95% CI −14.30 to 1.87; *I*²=84.2%; Figure S5 in Multimedia Appendix 5). These findings indicate limited robustness of the functional disability outcome.

For kinesiophobia, the leave-one-out analysis showed that the pooled result was sensitive to the omission of individual studies. After exclusion of a study by Karaduman and Ataş Balci [51], the pooled effect became statistically significant (MD −4.33, 95% CI −7.91 to −0.76; *P*=.03; Figure S6 in Multimedia Appendix 5). After excluding studies judged as having a high risk of bias [60], the pooled effect remained nonsignificant (MD −2.03, 95% CI −7.09 to 3.02; *I*²=84.6%; Figure S7 in Multimedia Appendix 5). These findings indicate limited robustness of the kinesiophobia outcome and suggest that this result should be interpreted cautiously.

### Small-Study Effects Assessment

The funnel plot for pain intensity showed some visual asymmetry, with smaller studies appearing unevenly distributed around the pooled effect estimate (Figure S8 in Multimedia Appendix 5). However, Egger’s regression test did not indicate statistically significant small-study effects (*t*_10_=−2.06; *P*=.07).

For functional disability, the funnel plot showed slight visual asymmetry (Figure S9 in Multimedia Appendix 5), but Egger’s regression test did not indicate statistically significant small-study effects (*t*_8_=−0.85; *P*=.42).

Given the substantial heterogeneity in the pooled analyses for pain intensity and functional disability (*I*²=90% and *I*²=83%, respectively), funnel plot asymmetry and Egger test results should be interpreted cautiously, as they may have been influenced by clinical and methodological heterogeneity. Overall, the current findings did not provide statistically significant evidence of small-study effects.

As only 6 (42.9%) studies were included for kinesiophobia, the Egger test was not performed for this outcome because of insufficient statistical power [50].

## Discussion

### Principal Findings

This systematic review and meta-analysis included 14 RCTs involving 794 adults with CNSLBP. Telerehabilitation was associated with statistically significant average reductions in pain intensity and functional disability compared with control conditions, whereas its effect on kinesiophobia was not statistically significant. However, these findings should be interpreted cautiously because the certainty of evidence was low or very low, heterogeneity was substantial across the main meta-analyses, and the 95% PIs crossed the null for all main outcomes.

The substantial heterogeneity limits confidence in the consistency and generalizability of the pooled estimates because random-effects summary estimates represent average effects across diverse clinical and intervention contexts, whereas PIs describe the expected distribution of true effects across settings [65,66]. The observed heterogeneity was likely related to differences in comparator intensity and content, delivery mode, therapist involvement, supervision level, intervention components, adherence support, and participant characteristics. As all PIs crossed the null, the magnitude and direction of effects may vary across clinical settings and intervention models. Therefore, statistical significance in the pooled mean effect should not be interpreted as evidence of consistent clinical benefit.

The exploratory subgroup findings were consistent with this interpretation. The apparent benefits of telerehabilitation were mainly observed in comparisons with minimal or nonactive controls, whereas no clear advantage was observed over active rehabilitation controls. This pattern suggests that part of the pooled effect may reflect the added value of receiving a structured rehabilitation program compared with little or no structured care, rather than a specific advantage of the remote mode of delivery over conventional active rehabilitation. Therefore, the observed effectiveness of telerehabilitation should be interpreted in relation to comparator intensity, intervention structure, supervision intensity, and clinical context.

### Interpretation and Comparison With Prior Work

This context-dependent pattern may help explain the inconsistent conclusions reported in previous reviews [26-29]. Earlier syntheses often pooled studies with markedly different comparator conditions, including wait-list or no-treatment controls, education or usual care, unsupervised home exercise, and structured face-to-face rehabilitation [27,29]. When such heterogeneous comparators are combined, the pooled estimate may address a mixture of different clinical questions, including whether telerehabilitation is superior to little or no structured rehabilitation and whether it provides added benefit over structured in-person rehabilitation. If pooled effects are disproportionately influenced by studies using low-intensity or minimal or nonactive controls, the apparent benefit of telerehabilitation may be overestimated. Therefore, considering comparator intensity in both the synthesis and interpretation is important for understanding the source and clinical meaning of the observed effects.

In this review, telerehabilitation showed more evident effects against minimal or nonactive controls, possibly because it provided more structured exercise guidance, feedback, and supervision than little or no intervention. In contrast, conventional face-to-face rehabilitation and other active treatments already include effective components, such as structured exercise training, therapist guidance, and supervision [13,67]. Therefore, when telerehabilitation was compared with active rehabilitation controls, no clear advantage was observed, which is broadly consistent with previous systematic reviews of digital health interventions in populations with chronic LBP [24,68]. Overall, the clinical value of telerehabilitation may lie less in replacing conventional active rehabilitation and more in supporting self-management, improving continuity of care, and extending access to rehabilitation services when face-to-face care is limited [69,70].

Telerehabilitation should be understood as a mode of rehabilitation delivery rather than a single standardized therapeutic intervention [71,72]. The included trials varied substantially in digital format, therapeutic content, therapist involvement, feedback mechanisms, and supervision intensity. Some interventions were mainly self-directed app-based or web-based programs [53,58,59], whereas others involved synchronous videoconferencing, telephone-supported instruction, virtual reality, or hybrid models combining in-person instruction with remote follow-up [51,54,57,62]. These models may differ not only in the technology used but also in treatment dose, individualization, exercise implementation and progression, adherence support, timeliness of feedback, and the extent to which therapists can monitor symptoms or correct exercise performance. As a result, trials labeled as telerehabilitation may represent quite different rehabilitation experiences for patients. These differences may have contributed to the varying subgroup effects observed for pain intensity and functional disability.

Exploratory subgroup analyses by delivery mode suggested that self-directed or asynchronous interventions showed more evident effects than therapist-supported or synchronous interventions for pain intensity and functional disability. Subgroup analyses by supervision level also suggested possible differences for pain intensity, although this pattern was not consistently observed across all outcomes. However, delivery mode, supervision level, and comparator intensity were closely interrelated. For example, self-directed or asynchronous interventions were more often compared with minimal or nonactive controls, whereas therapist-supported or synchronous interventions were more likely to be compared with active rehabilitation. Therefore, these subgroup differences may reflect the combined influence of intervention characteristics and comparator effects, rather than the independent effect of a specific delivery mode or supervision level. As delivery mode and comparator intensity were closely linked, these subgroup patterns cannot be attributed to delivery mode alone.

### Clinical Relevance and Kinesiophobia

Clinical relevance should be interpreted by distinguishing the pooled average effect from the expected distribution of true effects across settings. As pooled MDs describe the average intervention effect across the included studies, whereas PIs describe the range in which the true effect may be expected to fall in a new clinical setting, population, or intervention model, an average effect below the prespecified MCID threshold does not necessarily mean that clinically important benefit is unlikely in all settings [48].

For pain intensity, the pooled point estimate (MD −1.02) and its 95% CI (−1.78 to −0.26) did not reach the prespecified MCID threshold of 2.0 points [73,74], indicating that the average effect was statistically significant but below the threshold for clinically important improvement. However, the lower bound of the PI (−3.61) exceeded the MCID threshold in magnitude in the direction of benefit, suggesting that clinically important pain reduction may occur in some settings. At the same time, as the PI crossed the null (−3.61 to 1.51), little or no benefit may occur in other settings.

For functional disability, the pooled point estimate (MD −7.04) did not reach the MCID threshold of 10.0 points [74]. However, the lower bound of the 95% CI (−13.43) exceeded this threshold in magnitude in the direction of benefit, indicating that the average effect remains compatible with clinically important improvement, although the CI also included average effects smaller than the MCID. In addition, the lower bound of the PI (−27.97) suggested that clinically important functional improvement may occur in some settings. Nevertheless, because the PI also crossed the null (−27.97 to 13.91), the expected effect remains heterogeneous and uncertain across settings.

For kinesiophobia, the pooled average effect was not statistically significant, and the point estimate (MD −3.14) did not reach the MCID threshold of 5.5 points [75]. The 95% PI was wide and crossed the null (−15.04 to 8.47), indicating that the expected effect may range from clinically important improvement in some settings to little or no benefit in others. Together with the very low certainty of evidence, wide uncertainty, and sensitivity to the exclusion of individual studies, these findings indicate that the evidence for the kinesiophobia remains insufficient and unstable.

In subgroup analyses, the clinical relevance of telerehabilitation appeared more evident when compared with minimal or nonactive controls. In this subgroup, the average improvement in functional disability (MD −13.38) exceeded the MCID threshold of 10.0 points [74], whereas the average improvements in pain intensity and kinesiophobia (MD −1.77 and MD −5.20, respectively) approached but did not clearly exceed their corresponding thresholds of 2.0 and 5.5 points [73-75]. In contrast, when telerehabilitation was compared with active controls, none of the 3 average subgroup effects reached the corresponding MCID threshold, and the effect estimate for functional disability favored active rehabilitation. Thus, clinically important benefits may occur in some contexts, particularly compared with minimal or nonactive management, but the clinical relevance of telerehabilitation remains uncertain when compared with structured active rehabilitation.

Evidence for kinesiophobia remains insufficient in the overall analysis. The pooled average effect was not statistically significant, the point estimate did not reach the MCID threshold, both the CI and PI were wide and crossed the null, and the result was sensitive to the exclusion of individual studies. Although these wide intervals do not exclude clinically important improvement in some settings, the overall evidence remains insufficient and unstable. This limited robustness may reflect both the small evidence base and the complexity of kinesiophobia as a psychological and behavioral outcome. Within the biopsychosocial model of chronic pain, kinesiophobia is influenced not only by pain intensity but also by pain-related beliefs, fear avoidance behaviors, emotional distress, self-efficacy, and expectations about movement [76-78]. Therefore, improvement in kinesiophobia may require interventions that directly target cognitive and behavioral mechanisms, such as pain neuroscience education, cognitive behavioral strategies, graded activity, or graded exposure [79,80]. In this review, most telerehabilitation interventions were exercise-based or multicomponent rather than purely education-focused, and relatively few interventions included explicit components targeting fear avoidance or maladaptive pain beliefs. This may partly explain why the average effects of telerehabilitation appeared more evident for pain intensity and functional disability, whereas its effect on kinesiophobia remained unstable.

Differences between education-oriented and exercise-oriented components may also have contributed to heterogeneity. Exercise-based telerehabilitation may more directly affect pain and functional disability by increasing movement exposure, strength, motor control, and activity tolerance, whereas education- or behavior-oriented components may primarily target pain beliefs, fear avoidance, self-efficacy, and self-management [13,67,80]. However, many included interventions combined exercise, education, monitoring, and feedback; few studies were clearly dominated by education-focused approaches; and intervention components and implementation details were variably or incompletely reported. Therefore, this review could not isolate the independent contribution of each component or reliably compare education-based and exercise-based telerehabilitation as distinct intervention categories.

### Limitations

Several limitations should be acknowledged. First, although this review was registered in PROSPERO, the registration record underwent a major protocol amendment from the initial review question to telerehabilitation for CNSLBP. Therefore, the final review question should not be interpreted as fully prospectively registered in the same way as a review whose PICOS criteria, outcomes, and synthesis plan remained unchanged from the original registration. This weakens one of the main methodological safeguards usually associated with prospective registration, namely reducing the risk of selective review question formulation, eligibility decisions, outcome selection, or analysis planning [81,82]. To mitigate this concern, we explicitly reported the amendment history; described the final PICOS criteria and analytic decisions in detail; provided the main protocol deviations, potential implications, and corrective actions in Multimedia Appendix 1; listed excluded full-text studies with reasons; and interpreted post hoc subgroup analyses as exploratory rather than confirmatory. Nevertheless, these transparency measures cannot fully remove the limitations introduced by the major protocol amendment, and this issue should be considered when assessing the credibility and robustness of the findings.

Second, the certainty, robustness, and generalizability of the findings were limited by the small number of eligible trials, small sample sizes, substantial heterogeneity, and risk of bias in the included trials. In particular, one included trial enrolled only 8 participants [63], and several outcome-specific subgroup analyses included very few studies, which may have reduced the stability of the pooled and subgroup estimates. Most included trials were judged as having some concerns or high risk of bias. As blinding of participants and therapists is difficult in telerehabilitation and exercise-based trials [83], and because pain intensity, functional disability, and kinesiophobia were all patient-reported outcomes, the pooled estimates may have been influenced by treatment expectations, differential engagement or adherence, deviations from intended interventions, and outcome assessment related to awareness of group allocation [84].

Third, the subgroup analyses were exploratory. Comparator intensity, delivery mode, and supervision level were not mutually independent, and several subgroups included only a small number of studies. Formal meta-regression was not conducted because the number of eligible trials and outcome-specific comparisons was limited, which would make such analyses underpowered and potentially unstable [66]. Therefore, potential sources of heterogeneity could not be formally tested, and the subgroup analyses should be considered hypothesis-generating rather than confirmatory evidence that one telerehabilitation model is superior to another.

Finally, because the primary analyses preferentially pooled postintervention data, the long-term effects of telerehabilitation remain uncertain. This is particularly important for CNSLBP, which is often recurrent and requires sustained self-management rather than short-term symptom relief alone [12,85]. In addition, key implementation-related outcomes, including adherence, intervention fidelity, adverse events, safety monitoring, and economic outcomes, were inconsistently or sparsely reported across trials. These reporting gaps are important because the effectiveness, feasibility, and safety of telerehabilitation may depend on whether the intervention is delivered and used as intended [86]. Therefore, the available evidence remains insufficient to draw firm conclusions about long-term clinical effectiveness, implementation quality, and safety in routine care.

### Implications for Clinical Practice and Research

Despite these limitations, the findings have implications for rehabilitation delivery and health service planning. Telerehabilitation should not be interpreted as a universally superior replacement for structured active rehabilitation, but rather as a flexible delivery option or adjunctive strategy to support self-management, improve continuity of care, and extend access to rehabilitation services [71]. Telerehabilitation may therefore be useful for maintaining rehabilitation support between visits or after the initial assessment, including structured home exercise guidance, educational reinforcement, adherence feedback, and symptom self-monitoring when regular face-to-face rehabilitation is difficult to sustain [87,88]. Such an approach may be particularly relevant for patients living in remote areas, patients who have difficulty attending regular rehabilitation sessions, and health care settings with limited rehabilitation resources [87,89,90].

The economic value of telerehabilitation remains uncertain. Previous systematic reviews of musculoskeletal conditions have suggested that telerehabilitation may be associated with lower per-patient costs in some economic evaluations [23], and one included trial in this review also reported lower intervention costs for telerehabilitation than for outpatient rehabilitation [55]. These potential savings may be related to reduced travel, fewer facility-based visits, and lower time-related burdens for patients [91,92]. However, because economic outcomes were rarely reported and cost-related evidence varied across health care contexts [91], these findings should be interpreted cautiously.

For patients with prominent kinesiophobia or other psychological and behavioral barriers, remote exercise training alone may be insufficient because improvement in these outcomes may require interventions that directly address fear avoidance, maladaptive pain beliefs, self-efficacy, and engagement with activity [93,94]. Future telerehabilitation programs for CNSLBP should clarify whether educational content is used merely as supportive information or as an active therapeutic component. When fear avoidance is a major treatment target, programs may need to integrate psychologically informed strategies, such as pain neuroscience education, cognitive behavioral strategies, graded activity, or graded exposure [95].

Future trials should improve the reporting of treatment adherence and intervention fidelity. Across the included trials, adherence was assessed inconsistently using different indicators, such as session completion, digital engagement, logbook records, telephone follow-up, or platform-recorded activity [52-54,58,60]. Few trials clearly reported the actual exercise dose completed, intensity progression, therapist feedback, protocol deviations, or whether the intervention was delivered as intended. More standardized reporting of adherence and fidelity would help clarify whether observed effects reflect the telerehabilitation model itself or differences in actual treatment exposure and intervention implementation [86,96].

Finally, the applicability of the current evidence should be considered in light of the rapid development of digital rehabilitation technologies. Most included trials evaluated relatively simple telerehabilitation models, such as app-based exercise, video programs, telephone follow-up, virtual reality, or videoconference-based supervision. As digital rehabilitation increasingly incorporates wearable sensors, automated movement assessment, adaptive feedback, AI-supported monitoring, and hybrid remote–in-person care pathways [25,97], the current evidence may not fully represent the effectiveness, safety, adherence, or implementation feasibility of future digitally enhanced telerehabilitation models. Future trials should therefore evaluate not only clinical outcomes but also usability, engagement, safety monitoring, implementation feasibility, intervention fidelity, and cost-effectiveness, which may provide more systematic evidence for optimizing telerehabilitation delivery in contemporary digital care settings [98].

### Conclusions

The current evidence suggests that telerehabilitation may provide context-dependent benefits for adults with CNSLBP, particularly for improving pain intensity and functional disability when compared with minimal or nonactive controls. Although the pooled average effects for pain intensity and functional disability were below the prespecified MCID thresholds, the PIs suggested that clinically important benefits may occur in some settings, whereas little or no benefit may occur in others. No clear advantage was observed over active rehabilitation controls, and evidence for kinesiophobia remains insufficient and unstable. Given the substantial heterogeneity, PIs crossing the null, risk of bias, imprecision, and low or very low certainty of evidence, these findings should be interpreted cautiously. Telerehabilitation may be considered a flexible delivery option or adjunctive strategy to support self-management and improve access to rehabilitation services, especially when face-to-face care is limited. Future high-quality RCTs are needed to clarify which telerehabilitation models are effective, for whom, and under what clinical conditions.

## Supplementary material

10.2196/94835 Multimedia Appendix 1 Protocol deviations and corrective actions.

10.2196/94835 Multimedia Appendix 2 Search strategies for all databases.

10.2196/94835 Multimedia Appendix 3 Excluded full-text studies with reasons.

10.2196/94835 Multimedia Appendix 4 Subgroup analyses.

10.2196/94835 Multimedia Appendix 5 Sensitivity analyses and small-study effects assessment.

10.2196/94835 Checklist 1 PRISMA 2020 checklist, PRISMA 2020 for Abstracts checklist, and PRISMA-S checklist.

## Abbreviations

CNSLBP: chronic nonspecific low back pain
GRADE: Grading of Recommendations Assessment, Development and Evaluation
LBP: low back pain
MCID: minimal clinically important difference
MD: mean difference
PI: prediction interval
PICOS: population, intervention, comparator, outcomes, and study
PRISMA: Preferred Reporting Items for Systematic Reviews and Meta-Analyses
PRISMA-S: Preferred Reporting Items for Systematic Reviews and Meta-Analyses—Search Extension
RCT: randomized controlled trial
RoB 2.0: Risk of Bias 2.0 tool
