# Supplementary material for: Effectiveness of Telerehabilitation for Chronic Nonspecific Low Back Pain: Systematic Review and Meta-Analysis of Randomized Controlled Trials
Source: J Med Internet Res. 2026 Aug 10;28:e94835. doi: 10.2196/94835 (PMC13458355; doi:10.2196/94835)
Supplement: Supplementary file 1 [file jmir-v28-e94835-s001.docx]

**Protocol deviations and corrective actions**

| **Protocol item** | **Registered protocol / earlier version** | **Final review** | **Deviation or clarification** | **Potential implication** | **Corrective action** |
| --- | --- | --- | --- | --- | --- |
| PROSPERO registration and version history | The review was registered in PROSPERO under registration number CRD420251151727. The original record was made available on September 20, 2025. An amended record focusing on telerehabilitation for chronic nonspecific low back pain was made available on January 10, 2026. | The final review focuses on telerehabilitation for chronic nonspecific low back pain. | The registration record underwent a major amendment. The final review question should not be described as a fully prospective registration of the final review question. | This weakens the methodological protection usually provided by fully prospective registration, particularly in relation to selective review question formulation, eligibility decisions, outcome selection, and analysis planning. | The manuscript now reports the review as a registered review with a subsequent major protocol amendment. The version history and the nature of the amendment are explicitly described in the Methods section. |
| Review title and scope | The earlier PROSPERO version focused on cognitive functional therapy for chronic nonspecific low back pain. | The final manuscript focuses on telerehabilitation for chronic nonspecific low back pain. | The review scope was substantially changed from cognitive functional therapy to telerehabilitation. This represented a major protocol amendment. | The final review addresses a substantially different intervention question from the original record; therefore, the final scope should not be interpreted as unchanged from the original registration. | The Methods section now explicitly states that the registration record was substantially amended from cognitive functional therapy to telerehabilitation. |
| Review objective | The earlier objective was to evaluate the effectiveness of cognitive functional therapy compared with usual care or other active interventions in adults with chronic nonspecific low back pain. | The final objective is to evaluate the effects of telerehabilitation on pain intensity, functional disability, and kinesiophobia in adults with chronic nonspecific low back pain, and to explore whether these effects differed according to comparator intensity, delivery mode, and supervision level. | The objective was changed to match the amended review scope. | Changes in the objective may raise concern that the review question was refined after awareness of the available evidence. | The final objective was revised in the Introduction, and the Methods section was aligned with the revised objective through the PICOS framework, outcome definitions, and subgroup analysis plan. The review is no longer presented as a direct evaluation of cognitive functional therapy. |
| Population | Adults with chronic nonspecific low back pain were eligible. | Adults with chronic nonspecific low back pain were eligible. Chronicity was defined as low back pain lasting at least 12 weeks or 3 months. Studies involving serious or specific spinal pathology or predominant radicular pain were excluded. | No major deviation. The final manuscript provides a more detailed operational definition of chronic nonspecific low back pain. | Clarification improves reproducibility and reduces the risk of indirectness from including specific low back pain conditions or predominant radicular pain. | The eligibility criteria were clarified using the PICOS framework. |
| Intervention definition | The earlier version included cognitive functional therapy alone or in combination with another active treatment. | Telerehabilitation was defined as the remote delivery or support of rehabilitation-related interventions through digital or communication technologies, including web-based platforms, mobile apps, video-based programs, virtual reality systems, telephone or video-call support, and hybrid models combining initial in-person instruction with remote follow-up. | The intervention definition was substantially changed. | The expanded intervention scope increases clinical and methodological heterogeneity and requires explicit boundaries between telerehabilitation and broader eHealth/mHealth tools or passive remote monitoring. | A structured intervention classification table was added to describe telerehabilitation modality, delivery mode, supervision level, feedback/monitoring, and comparator characteristics. |
| Comparator framework | The earlier protocol referred to usual care or other active interventions. | Comparators were classified as active controls or minimal/non-active controls according to treatment content, supervision intensity, and degree of individualization. | The comparator framework was refined because the included trials used heterogeneous control conditions. | Comparator classification may influence subgroup estimates and interpretation of effectiveness, particularly because active and minimal/non-active controls differ substantially in treatment intensity and individualization. | Operational criteria for comparator classification were added to the Methods section. A table was added to justify the classification of each comparator. |
| Outcomes | The earlier record listed pain intensity and body function as key outcomes and also referred to quality of life and adverse events in relation to certainty assessment. | The final meta-analysis synthesized pain intensity, functional disability, and kinesiophobia. | The outcome set was refined. Quality of life and adverse events were not meta-analyzed because they were not consistently reported across eligible studies. Kinesiophobia was included because it was clinically relevant to chronic nonspecific low back pain rehabilitation and aligned with the amended review objective; it was synthesized quantitatively only when sufficient comparable data were available. | Refining the outcome set may raise concern about selective outcome synthesis, particularly if outcomes were included because they were sufficiently reported across eligible trials. | The final outcomes and measurement instruments were specified in the Methods section. Outcomes not suitable for quantitative synthesis were not presented as pooled outcomes. |
| Study design | Randomized controlled trials were eligible. | Randomized controlled trials were eligible. | No deviation. | Retaining RCTs limits design-related bias and preserves alignment with the registered study-design criterion. | The final eligibility criteria retained randomized controlled trials only. |
| Language eligibility | The PROSPERO record specified that studies published in English or Chinese would be eligible. | Studies published in English or Chinese were eligible. Chinese databases were searched as part of the final search strategy. | No deviation in language eligibility in the revised manuscript. The initial reporting of language eligibility required clarification. | Restricting eligibility to English and Chinese may miss studies in other languages; however, searching Chinese databases improves coverage of Chinese-language literature. | The Methods section was revised to state that studies published in English or Chinese were eligible, and the Search Strategy section was revised to include Chinese databases. |
| Information sources | The PROSPERO record listed major databases including CENTRAL/Cochrane Library, Embase, PubMed, SCI/SSCI, VIP, and Wanfang, and also mentioned additional methods such as citation searching, reference checking, trial registries, conference abstracts, and contacting authors or experts where necessary. | The final review searched PubMed, Embase, Web of Science, PEDro, CENTRAL, CNKI, Wanfang, VIP, and CBM from inception to November 2, 2025, with a final update on May 8, 2026. | Information sources were modified and expanded. Web of Science was used to operationalize SCI/SSCI searching, and PEDro, CNKI, and CBM were added to improve coverage. | Because supplementary search methods were not used systematically in the final review, unpublished, ongoing, or grey-literature studies may have been missed. | The Search Strategy section was revised to report the final database list. Complete search strategies for all databases are provided in Multimedia Appendix 2. |
| Search date | The PROSPERO record did not impose a restriction on publication date. | The final systematic search was conducted from inception to November 2, 2025, with a final update on May 8, 2026. | The final manuscript reports both the original search date and the final update date for reproducibility. | The final search update improves currency and reduces the likelihood that recently published eligible trials were missed. | The Methods section and search strategy appendix were revised to report the search period as from inception to November 2, 2025, with a final update on May 8, 2026. The PRISMA flow diagram was updated to reflect the final search results. |
| Search strategy | The earlier protocol described searches using terms related to the original intervention focus. | The final search strategy combined terms related to low back pain, telerehabilitation or digital health, and randomized controlled trials. Controlled vocabulary terms such as MeSH and EMTREE were used where applicable. | The search strategy was revised to match the amended review question and intervention definition. | Changing search terms to match the amended question was necessary, but it means the final search strategy differed from the original intervention-focused strategy. | The final search strategy was developed according to PRISMA-S and is reported in detail in Multimedia Appendix 2. |
| Study selection | At least two reviewers were planned to independently screen records, with disagreements resolved through discussion or adjudication. | Two reviewers independently screened studies, and disagreements were resolved by discussion or by a third reviewer. | No major deviation. | Independent screening reduces the risk of arbitrary eligibility decisions and improves reproducibility. | The final Methods section reports the independent screening process and disagreement resolution procedure. |
| Data extraction | At least two reviewers were planned to independently extract data, with disagreements resolved through discussion or adjudication. | Two reviewers independently extracted data using a standardized data extraction form, and a third reviewer was consulted when necessary. | No major deviation. | Independent extraction reduces data extraction errors and subjective extraction decisions. | The final Methods section reports the data extraction process and extracted variables. |
| Intervention characteristics extracted | The earlier protocol did not fully specify detailed telerehabilitation characteristics because the initial intervention focus was cognitive functional therapy. | The final review extracted telerehabilitation modality, delivery mode, intervention components, duration and frequency, supervision level, feedback or monitoring methods, and comparator characteristics. | Additional intervention-related variables were extracted to address the clinical heterogeneity of telerehabilitation models. | Additional variables were needed to interpret heterogeneity, but classification may still involve some subjectivity when reporting in primary trials was incomplete. | A structured intervention and comparator classification table was added to improve transparency and interpretability. |
| Risk-of-bias assessment | The PROSPERO record specified use of the Cochrane RoB 2 tool. | The final review used the Cochrane Risk of Bias 2.0 tool. | No deviation. | Using the planned tool supports consistency with the registered methodological quality assessment. | The Methods and Results sections report RoB 2.0 assessment and summarize the main sources of bias. |
| Certainty-of-evidence assessment | The PROSPERO record planned certainty assessment using GRADE. | The final review evaluated the certainty of evidence using GRADE for the main outcomes and comparator-based subgroup outcomes where appropriate. | No major deviation. GRADE reporting was expanded. | Expanded GRADE reporting aligns the strength of conclusions with the certainty of evidence, but it also highlights reduced confidence due to heterogeneity, risk of bias, imprecision, and small sample sizes. | GRADE tables were added, and the interpretation of findings was revised in light of low or very low certainty of evidence. |
| Effect measures | The earlier protocol planned use of mean difference or standardized mean difference for continuous outcomes and risk ratio for dichotomous outcomes where applicable. | The final review used mean differences with 95% confidence intervals for continuous outcomes measured on harmonized scales. VAS scores on a 0-100 scale were rescaled to a 0-10 metric and pooled with NRS scores. Standardized mean differences were planned if scales could not be harmonized. | No major deviation for continuous outcomes. Dichotomous outcomes were not synthesized because the eligible outcomes for meta-analysis were continuous. | Not synthesizing dichotomous outcomes limits conclusions about safety, adverse events, or other binary outcomes when these data were insufficiently reported. | The Statistical Analysis section explains scale harmonization and effect measure selection. |
| Statistical model selection | The earlier protocol indicated use of a random-effects model when substantial heterogeneity was detected and a fixed-effect model otherwise. | The final review used random-effects models for all meta-analyses because clinical and methodological heterogeneity was expected across telerehabilitation modalities, supervision levels, intervention durations, and comparator intensities. | The model selection approach was changed from heterogeneity-statistic-based selection to a conceptual random-effects approach. | Using random-effects models throughout is appropriate for expected clinical and methodological heterogeneity but generally leads to wider intervals and more cautious inference. | The Statistical Analysis section explains that random-effects models were selected based on expected clinical and methodological heterogeneity rather than I² alone. |
| Random-effects estimator and adjustment | The earlier protocol did not specify the restricted maximum likelihood estimator or Hartung-Knapp-Sidik-Jonkman adjustment. | Random-effects models were fitted using the restricted maximum likelihood estimator with Hartung-Knapp-Sidik-Jonkman adjustment. | The statistical method was refined. | This refinement may affect confidence interval width compared with conventional methods and generally provides more conservative inference when the number of studies is small. | The Statistical Analysis section now reports REML and Hartung-Knapp-Sidik-Jonkman adjustment. |
| Prediction intervals | Prediction intervals were not specified in the earlier protocol. | Ninety-five percent prediction intervals were calculated for the main random-effects meta-analyses where statistically estimable and clinically interpretable. | Prediction intervals were added after methodological reconsideration of heterogeneity. | Adding prediction intervals shifts interpretation beyond the average pooled effect to the expected distribution of effects across settings, emphasizing uncertainty when intervals cross the null. | The Methods and Results now distinguish between the average pooled effect and the expected distribution of true effects across settings. Prediction intervals were not calculated for subgroup analyses with limited study numbers. |
| Multi-arm trials and double counting | The earlier protocol did not fully specify handling of multi-arm trials. | For multi-arm trials, all potentially eligible arms were assessed against the predefined PICOS criteria. When multiple telerehabilitation arms were eligible for an outcome-specific meta-analysis, the comparison most closely aligned with the prespecified PICO was selected, and double-counting of participants or shared comparator groups was avoided. | The handling of multi-arm trials was clarified. | Clarification reduces unit-of-analysis error, but some otherwise eligible arms were not pooled when including them would have double-counted participants or shared comparators. | The Statistical Analysis section now explicitly states how multi-arm trials were handled to avoid unit-of-analysis errors. |
| Subgroup analyses | The earlier protocol mentioned subgroup analyses to explore heterogeneity but did not fully specify the final subgroup categories. | Exploratory subgroup analyses were conducted according to comparator type, delivery mode, and supervision level when at least two studies were available in each relevant subgroup. | Subgroup categories were refined and treated as exploratory. | Because these subgroup categories were not fully prespecified and were not mutually independent, subgroup findings should be interpreted as hypothesis-generating rather than confirmatory. | The Methods section explicitly states that these subgroup analyses were exploratory and were not used to establish definitive superiority of one telerehabilitation model over another. |
| Meta-regression | Meta-regression was not clearly prespecified. | Meta-regression was not performed. | Not applicable; meta-regression was not prespecified and was not feasible. | Potential sources of heterogeneity could not be formally tested using meta-regression; therefore, explanations for heterogeneity remain exploratory. | Meta-regression was not conducted because the number of included studies per outcome and per potential covariate was limited, which would make the analysis underpowered and prone to overfitting. |
| Sensitivity analyses | The earlier protocol planned sensitivity analyses where appropriate. | The final review performed leave-one-out analyses and additional sensitivity analyses excluding studies with very small sample sizes and studies judged to be at high risk of bias. | Sensitivity analyses were expanded. | Expanded sensitivity analyses improve robustness assessment, but analyses not fully prespecified should be interpreted as supportive rather than definitive. | Sensitivity analyses were added to evaluate the robustness of findings in relation to small sample sizes and risk of bias. |
| Small-study effects | The earlier protocol mentioned funnel plots and statistical tests when at least 10 studies were available. | The final review used funnel plots and Egger regression tests when at least 10 studies were available and interpreted these as indicators of small-study effects rather than definitive evidence of publication bias. | The terminology and interpretation were refined to avoid equating small-study effects with definitive publication bias. | Funnel plots and Egger tests may be underpowered or influenced by heterogeneity and should not be interpreted as definitive evidence for or against publication bias. | The Results and Discussion were revised to distinguish small-study effects from publication bias and to avoid overinterpreting funnel plot asymmetry. |
| Interpretation of findings | The earlier protocol did not fully specify how findings would be interpreted in relation to comparator intensity, prediction intervals, risk of bias, and GRADE. | The final manuscript interprets findings in light of heterogeneity, comparator intensity, prediction intervals, risk of bias, small-study effects, and GRADE certainty. | Interpretation was refined to avoid overstatement of effectiveness. | The conclusions are more cautious and should be understood as context-dependent rather than as evidence of a consistent advantage of telerehabilitation across settings. | The Discussion and Conclusions were revised to emphasize context-dependent effects, low or very low certainty of evidence, and lack of clear advantage over active controls. |
