# Supplementary material for: Effectiveness of Telerehabilitation for Chronic Nonspecific Low Back Pain: Systematic Review and Meta-Analysis of Randomized Controlled Trials
Source: J Med Internet Res. 2026 Aug 10;28:e94835. doi: 10.2196/94835 (PMC13458355; doi:10.2196/94835)
Supplement: Supplementary file 2 [file jmir-v28-e94835-s002.docx]

| **1. Search strategy for PubMed <May 8, 2026>** | |
| --- | --- |
| #1 | ((((((((((((((((((((((((((((((((((((((((telemedicine[Title/Abstract]) OR (telehealth[Title/Abstract])) OR (tele health[Title/Abstract])) OR (telecare[Title/Abstract])) OR (tele care[Title/Abstract])) OR (telehealthcare[Title/Abstract])) OR (telehealth care[Title/Abstract])) OR (tele healthcare[Title/Abstract])) OR (tele health care[Title/Abstract])) OR (electronic health[Title/Abstract])) OR (ehealth[Title/Abstract])) OR (mobile health[Title/Abstract])) OR (mhealth[Title/Abstract])) OR (m health[Title/Abstract])) OR (mobile medicine[Title/Abstract])) OR (home-based[Title/Abstract])) OR (phone[Title/Abstract])) OR (telephone[Title/Abstract])) OR (cellular phone[Title/Abstract])) OR (cell phone[Title/Abstract])) OR (smart phone[Title/Abstract])) OR (smartphone[Title/Abstract])) OR (mobile phone[Title/Abstract])) OR (apps[Title/Abstract])) OR (application program[Title/Abstract])) OR (application software[Title/Abstract])) OR (mobile application[Title/Abstract])) OR (mobile technology[Title/Abstract])) OR (SMS[Title/Abstract])) OR (internet-based[Title/Abstract])) OR (internet based[Title/Abstract])) OR (web-based[Title/Abstract])) OR (computer-based[Title/Abstract])) OR (computer based[Title/Abstract])) OR (videoconferencing[Title/Abstract])) OR (video conferencing[Title/Abstract])) OR (wireless[Title/Abstract])) OR (mobile tablet[Title/Abstract])) OR (tablet computer[Title/Abstract])) OR (iPad[Title/Abstract])) OR (iPhone[Title/Abstract]) 396053 |
| #2 | "Telemedicine"[Mesh] 57368 |
| #3 | #1 OR #2 411368 |
| #4 | (((((((((((((((((((((((((((((Back Pain, Low[Title/Abstract]) OR (Back Pains, Low[Title/Abstract])) OR (Low Back Pains[Title/Abstract])) OR (Pain, Low Back[Title/Abstract])) OR (Pains, Low Back[Title/Abstract])) OR (Low Back Ache[Title/Abstract])) OR (Ache, Low Back[Title/Abstract])) OR (Aches, Low Back[Title/Abstract])) OR (Back Ache, Low[Title/Abstract])) OR (Back Aches, Low[Title/Abstract])) OR (Low Back Aches[Title/Abstract])) OR (Low Backache[Title/Abstract])) OR (Backache, Low[Title/Abstract])) OR (Backaches, Low[Title/Abstract])) OR (Low Backaches[Title/Abstract])) OR (Lower Back Pain[Title/Abstract])) OR (Back Pain, Lower[Title/Abstract])) OR (Back Pains, Lower[Title/Abstract])) OR (Lower Back Pains[Title/Abstract])) OR (Pain, Lower Back[Title/Abstract])) OR (Pains, Lower Back[Title/Abstract])) OR (Lumbago[Title/Abstract])) OR (Low Back Pain, Mechanical[Title/Abstract])) OR (Mechanical Low Back Pain[Title/Abstract])) OR (Low Back Pain, Posterior Compartment[Title/Abstract])) OR (Low Back Pain, Postural[Title/Abstract])) OR (Postural Low Back Pain[Title/Abstract])) OR (Low Back Pain, Recurrent[Title/Abstract])) OR (Recurrent Low Back Pain[Title/Abstract])) 55154 |
| #5 | ("Low Back Pain"[Mesh]) 30346 |
| #6 | #4 OR #5 60843 |
| #7 | ((randomized controlled trial[Title/Abstract]) OR (randomized[Title/Abstract])) OR (placebo[Title/Abstract]) 956876 |
| #8 | #3 AND #6 AND #7 352 |
| **2. Search strategy for Web of Science <May 8, 2026>** | |
| #1 | TS=(telemedicine OR telehealth OR tele health OR telecare OR tele care OR telehealthcare OR telehealth care OR tele healthcare OR tele health care OR electronic health OR ehealth OR e health OR mobile health OR mhealth OR m health OR mobile medicine OR distance OR remotely delivered OR phone OR telephone OR cellular phone OR cell phone OR smart phone OR smartphone OR mobile phone OR apps OR application program OR application software OR mobile application OR mobile applications OR mobile communication OR mobile technology OR SMS OR messaging OR text messaging OR texting OR internet OR internet-based OR internet based OR online OR web-based OR web based OR webbased OR computer-based OR computer based OR wireless OR wireless communication OR videoconferencing OR video conferencing OR mobile tablet OR tablet computer OR tablet device OR iPad OR iPhone ) 2503776 |
| #2 | TS=(Back Pain, Low OR Back Pains, Low OR Low Back Pains OR Pain, Low Back OR Pains, Low Back OR Low Back Ache OR Ache, Low Back OR Aches, Low Back OR Back Ache, Low OR Back Aches, Low OR Low Back Aches OR Low Backache OR Backache, Low OR Backaches, Low OR Low Backaches OR Lower Back Pain OR Back Pain, Lower OR Back Pains, Lower OR Lower Back Pains OR Pain, Lower Back OR Pains, Lower Back OR Lumbago OR Low Back Pain, Mechanical OR Mechanical Low Back Pain OR Low Back Pain, Posterior Compartment OR Low Back Pain, Postural OR Postural Low Back Pain OR Low Back Pain) 49118 |
| #3 | TS=(randomized controlled trial OR randomized OR placebo) 876503 |
| #4 | #1 AND #2 AND #3 1458 |
| **3. Search strategy for Embase<May 8, 2026>** | |
| #1 | 'backache'/exp 168846 |
| #2 | 'back pain, low':ab,ti OR 'back pains, low':ab,ti OR 'low back pains':ab,ti OR 'pain, low back':ab,ti OR 'pains, low back':ab,ti OR 'low back ache':ab,ti OR 'ache, low back':ab,ti OR 'aches, low back':ab,ti OR 'back ache, low':ab,ti OR 'back aches, low':ab,ti OR 'low back aches':ab,ti OR 'low backache':ab,ti OR 'backache, low':ab,ti OR 'backaches, low':ab,ti OR 'low backaches':ab,ti OR 'lower back pain':ab,ti OR 'back pain, lower':ab,ti OR 'back pains, lower':ab,ti OR 'lower back pains':ab,ti OR 'pain, lower back':ab,ti OR 'pains, lower back':ab,ti OR 'lumbago':ab,ti OR 'low back pain, mechanical':ab,ti OR 'mechanical low back pain':ab,ti OR 'low back pain, posterior compartment':ab,ti OR 'low back pain, postural':ab,ti OR 'postural low back pain':ab,ti OR 'low back pain, recurrent':ab,ti OR 'recurrent low back pain':ab,ti 11867 |
| #3 | #1 OR #2 171013 |
| #4 | 'telemedicine'/exp 97847 |
| #5 | 'telemedicine':ab,ti OR 'telehealth':ab,ti OR 'tele health':ab,ti OR 'telecare':ab,ti OR 'tele care':ab,ti OR 'telehealthcare':ab,ti OR 'telehealth care':ab,ti OR 'tele healthcare':ab,ti OR 'tele health care':ab,ti OR 'electronic health':ab,ti OR 'ehealth':ab,ti OR 'e health':ab,ti OR 'mobile health':ab,ti OR 'mhealth':ab,ti OR 'm health':ab,ti OR 'mobile medicine':ab,ti OR 'home-based':ab,ti OR 'home based':ab,ti OR 'distance':ab,ti OR 'remotely delivered':ab,ti OR 'self-management':ab,ti OR 'self management':ab,ti OR 'phone':ab,ti OR 'telephone':ab,ti OR 'cellular phone':ab,ti OR 'cell phone':ab,ti OR 'smart phone':ab,ti OR 'smartphone':ab,ti OR 'mobile phone':ab,ti OR 'apps':ab,ti OR 'application program':ab,ti OR 'application software':ab,ti OR 'mobile application':ab,ti OR 'mobile applications':ab,ti OR 'mobile communication':ab,ti OR 'mobile technology':ab,ti OR 'sms':ab,ti OR 'messaging':ab,ti OR 'text messaging':ab,ti OR 'texting':ab,ti OR 'internet':ab,ti OR 'internet-based':ab,ti OR 'internet based':ab,ti OR 'online':ab,ti OR 'web-based':ab,ti OR 'web based':ab,ti OR 'webbased':ab,ti OR 'computer-based':ab,ti OR 'computer based':ab,ti OR 'wireless':ab,ti OR 'wireless communication':ab,ti OR 'videoconferencing':ab,ti OR 'video conferencing':ab,ti OR 'mobile tablet':ab,ti OR 'tablet computer':ab,ti OR 'tablet device':ab,ti OR 'ipad':ab,ti OR 'iphone':ab,ti 1484435 |
| #6 | #4 OR #5 1518114 |
| #7 | 'randomized controlled trial'/exp 1200711 |
| #8 | 'randomized controlled trial':ab,ti OR 'randomized':ab,ti OR 'placebo':ab,ti OR 'rct':ab,ti 1625816 |
| #9 | #7 OR #8 1972227 |
| #10 | #3 AND #6 AND #9 1532 |
| **4. Search strategy for Cochrane Library <May 8, 2026>** | |
| #1 | (telemedicine):ab,ti,kw OR (telehealth):ab,ti,kw OR (tele health):ab,ti,kw OR (telecare):ab,ti,kw OR (tele care):ab,ti,kw OR (telehealthcare):ab,ti,kw OR (telehealth care):ab,ti,kw OR (tele healthcare):ab,ti,kw OR (tele health care):ab,ti,kw OR (electronic health):ab,ti,kw OR (ehealth):ab,ti,kw OR (e health):ab,ti,kw OR (mobile health):ab,ti,kw OR (mhealth):ab,ti,kw OR (m health):ab,ti,kw OR (mobile medicine):ab,ti,kw OR (distance):ab,ti,kw OR (remotely delivered):ab,ti,kw OR (phone):ab,ti,kw OR (telephone):ab,ti,kw OR (cellular phone):ab,ti,kw OR (cell phone):ab,ti,kw OR (smart phone):ab,ti,kw OR (smartphone):ab,ti,kw OR (mobile phone):ab,ti,kw OR (apps):ab,ti,kw OR (application program):ab,ti,kw OR (application software):ab,ti,kw OR (mobile application):ab,ti,kw OR (mobile applications):ab,ti,kw OR (mobile communication):ab,ti,kw OR (mobile technology):ab,ti,kw OR (SMS):ab,ti,kw OR (messaging):ab,ti,kw OR (text messaging):ab,ti,kw OR (texting):ab,ti,kw OR (internet):ab,ti,kw OR (internet-based):ab,ti,kw OR (internet based):ab,ti,kw OR (online):ab,ti,kw OR (web-based):ab,ti,kw OR (web based):ab,ti,kw OR (webbased):ab,ti,kw OR (computer-based):ab,ti,kw OR (computer based):ab,ti,kw OR (wireless):ab,ti,kw OR (wireless communication):ab,ti,kw OR (videoconferencing):ab,ti,kw OR (video conferencing):ab,ti,kw OR (mobile tablet):ab,ti,kw OR (tablet computer):ab,ti,kw OR (tablet device):ab,ti,kw OR (iPad):ab,ti,kw OR (iPhone):ab,ti,kw 235958 |
| #2 | Telemedicine 8647 |
| #3 | #1 OR #2 236141 |
| #4 | Low Back Pain 19928 |
| #5 | (Back Pain, Low):ab,ti,kw OR (Back Pains, Low):ab,ti,kw OR (Low Back Pains):ab,ti,kw OR (Pain, Low Back):ab,ti,kw OR (Pains, Low Back):ab,ti,kw OR (Low Back Ache):ab,ti,kw OR (Ache, Low Back):ab,ti,kw OR (Aches, Low Back):ab,ti,kw OR (Back Ache, Low):ab,ti,kw OR (Back Aches, Low):ab,ti,kw OR (Low Back Aches):ab,ti,kw OR (Low Backache):ab,ti,kw OR (Backache, Low):ab,ti,kw OR (Backaches, Low):ab,ti,kw OR (Low Backaches):ab,ti,kw OR (Lower Back Pain):ab,ti,kw OR (Back Pain, Lower):ab,ti,kw OR (Back Pains, Lower):ab,ti,kw OR (Lower Back Pains):ab,ti,kw OR (Pain, Lower Back):ab,ti,kw OR (Pains, Lower Back):ab,ti,kw OR (Lumbago):ab,ti,kw OR (Low Back Pain, Mechanical):ab,ti,kw OR (Mechanical Low Back Pain):ab,ti,kw OR (Low Back Pain, Posterior Compartment):ab,ti,kw OR (Low Back Pain, Postural):ab,ti,kw OR (Postural Low Back Pain):ab,ti,kw OR (Low Back Pain, Recurrent):ab,ti,kw OR (Recurrent Low Back Pain):ab,ti,kw 20664 |
| #6 | #4 OR #5 22528 |
| #7 | (randomized controlled trial):ab,ti,kw OR ( randomized):ab,ti,kw OR ( placebo):ab,ti,kw OR (RCT):ab,ti,kw 1446279 |
| #8 | #3 AND #6 AND #7 1934 |
| **5. Search strategy for PEDro <May 8, 2026>** | |
| #1 | The PEDro database was searched using the Advanced Search interface. The following fields were applied:   - Abstract & Title: not used - Therapy: no appropriate value in this field - Problem: pain - Body Part: lumbar spine, sacro-iliac joint or pelvis - Method: clinical trial - Published Since: not restricted - Score of at least: not restricted   Records identified:119 |
| **6. Search strategy for CBM <May 8, 2026>** | |
| #1 | "腰痛"[不加权:扩展] 8339 |
| #2 | "腰痛"[常用字段:智能] OR "下腰痛"[常用字段:智能] OR "慢性腰痛"[常用字段:智能] OR "慢性非特异性腰痛"[常用字段:智能] OR "慢性下腰痛"[常用字段:智能] OR "慢性非特异性下腰痛"[常用字段:智能] OR "下背痛"[常用字段:智能] OR "慢性下背痛"[常用字段:智能] OR "慢性非特异性下背痛"[常用字段:智能]  30832 |
| #3 | (#1) OR (#2) 30832 |
| #4 | "远程康复"[不加权:扩展] 202 |
| #5 | "远程康复"[常用字段:智能] OR "远程医疗"[常用字段:智能] OR "在线康复"[常用字段:智能] OR "互联网康复"[常用字段:智能] OR "移动健康"[常用字段:智能] OR "数字健康"[常用字段:智能] OR "APP"[常用字段:智能] OR "手机应用"[常用字段:智能] OR "虚拟现实"[常用字段:智能] 18248 |
| #6 | "随机对照试验"[不加权:扩展] 199961 |
| #7 | "随机对照试验"[常用字段:智能] OR "随机对照实验"[常用字段:智能] OR "随机对照"[常用字段:智能] OR "随机"[常用字段:智能] OR "随机对照研究"[常用字段:智能] OR "RCT"[常用字段:智能] 2157832 |
| #8 | (#4) OR (#5) 18248 |
| #9 | (#6) OR (#7) 2157832 |
| #10 | (#3) AND (#9) AND (#8) 6 |
| **7. Search strategy for CNKI <May 8, 2026>** | |
| 1 | (主题：腰痛) OR (主题：下腰痛) OR (主题：慢性腰痛) OR (主题：慢性非特异性腰痛) OR (主题：慢性下腰痛) OR (主题：慢性非特异性下腰痛) OR (主题：下背痛) OR (主题：慢性下背痛) OR (主题：慢性非特异性下背痛) AND (主题：远程康复) OR (主题：远程医疗) OR (主题：在线康复) OR (主题：互联网康复) OR (主题：移动健康) OR (主题：数字健康) OR (主题：APP) OR (主题：VR) OR (主题：虚拟现实) OR (主题：手机应用) AND (摘要：随机对照试验(精确)) OR (摘要：随机对照实验(精确)) OR (摘要：随机对照研究(精确)) OR (摘要：RCT(精确)) OR (摘要：随机对照(精确)) OR (摘要：随机(精确))  Records identified: 239 |
| **8. Search strategy for Wanfang <May 8, 2026>** | |
| 1 | 主题:(腰痛 OR 下腰痛 OR 慢性腰痛 OR 慢性非特异性腰痛 OR 慢性下腰痛 OR 慢性非特异性下腰痛 OR 下背痛 OR 慢性下背痛 OR 慢性非特异性下背痛) and 主题:(远程康复 OR 远程医疗 OR 在线康复 OR 互联网康复 OR 移动健康 OR 数字健康 OR APP OR VR OR 虚拟现实 OR 手机应用) and 主题:(随机 OR 随机对照 OR 随机对照试验 OR 随机对照研究 OR 随机对照实验)  Records identified: 61 |
| **9. Search strategy for VIP <May 8, 2026>** | |
| 1 | 主题:(腰痛 OR 下腰痛 OR 慢性腰痛 OR 慢性非特异性腰痛 OR 慢性下腰痛 OR 慢性非特异性下腰痛 OR 下背痛 OR 慢性下背痛 OR 慢性非特异性下背痛) and 主题:(远程康复 OR 远程医疗 OR 在线康复 OR 互联网康复 OR 移动健康 OR 数字健康 OR APP OR VR OR 虚拟现实 OR 手机应用) and 主题:(随机 OR 随机对照 OR 随机对照试验 OR 随机对照研究 OR 随机对照实验)  Records identified: 5 |
