# Supplementary material for: Effectiveness of Telerehabilitation for Chronic Nonspecific Low Back Pain: Systematic Review and Meta-Analysis of Randomized Controlled Trials
Source: J Med Internet Res. 2026 Aug 10;28:e94835. doi: 10.2196/94835 (PMC13458355; doi:10.2196/94835)
Supplement: Supplementary file 3 [file jmir-v28-e94835-s003.docx]

| **No.** | **First author, year** | **Study title** | **Reason for exclusion** |
| --- | --- | --- | --- |
| 1 | Rothbaum et al  2023 | A pilot randomized controlled trial of virtual reality delivered relaxation for chronic low back pain | Ineligible population |
| 2 | Eccleston et al  2022 | A prospective, double-blind, pilot, randomized, controlled trial of an “embodied” virtual reality intervention for adults with low back pain | Ineligible population |
| 3 | Cimarras-Otal et al  2020 | Adapted exercises versus general exercise recommendations on chronic low back pain in industrial workers: A randomized control pilot study | Ineligible population |
| 4 | Marcuzzi et al  2023 | Effect of an artificial intelligence-based self-management app on musculoskeletal health in patients with neck and/or low back pain referred to specialist care: a randomized clinical trial | Ineligible population |
| 5 | Koppenaal et al  2023 | Effectiveness and Cost-Effectiveness of a Stratified Blended Physiotherapy Intervention Compared With Face-to-Face Physiotherapy in Patients With Nonspecific Low Back Pain: Cluster Randomized Controlled Trial | Ineligible intervention |
| 6 | Mesa-Castrillon et al  2024 | Effectiveness of an eHealth-delivered program to EMpower People with musculoskeletal pain in Rural Australia (EMPoweR): a randomised controlled trial. | Ineligible population |
| 7 | Fanuscu et al  2024 | Effects of Clinic-based and Telerehabilitation-based Motor Control Exercises in Individuals with Chronic Low-back Pain A Randomized Controlled Trial With 3-Month Follow-up | Ineligible population |
| 8 | Yeh et al  2020 | Efficacy of Electronic Acupuncture Shoes for Chronic Low Back Pain: Double-Blinded Randomized Controlled Trial | Ineligible intervention |
| 9 | Sanabria-Mazo et al  2023 | Efficacy of Videoconference Group Acceptance and Commitment Therapy (ACT) and Behavioral Activation Therapy for Depression (BATD) for Chronic Low Back Pain (CLBP) Plus Comorbid Depressive Symptoms: A Randomized Controlled Trial (IMPACT Study) | Ineligible population |
| 10 | Thomas et al  2016 | Feasibility and Safety of a Virtual Reality Dodgeball Intervention for Chronic Low Back Pain: A Randomized Clinical Trial | Ineligible intervention |
| 11 | Alten Blaskowitz et al  2024 | Impact of the digital health application ViViRA on spinal mobility, physical function, quality of life and pain perception in spondyloarthritides patients: a randomized controlled trial | Ineligible population |
| 12 | Amorim et al  2019 | Integrating Mobile-health, health coaching, and physical activity to reduce the burden of chronic low back pain trial (IMPACT): a pilot randomised controlled trial | Ineligible population |
| 13 | Licciardone et al  2022 | Optimizing chronic pain management through patient engagement with quality of life measures: a randomized controlled trial | Ineligible outcomes |
| 14 | Jamison et al  2019 | Outcome of a High Frequency Transcutaneous Electrical Nerve Stimulator (hfTENS) Device for Low Back Pain: A Randomized Controlled Trial | Ineligible intervention |
| 15 | Nambi et al  2021 | Radiological (Magnetic Resonance Image and Ultrasound) and biochemical effects of virtual reality training on balance training in football players with chronic low back pain: A randomized controlled study | Ineligible outcomes |
| 16 | Chhabra et al  2018 | Smartphone app in self‐management of chronic low back pain: a randomized controlled trial | Ineligible population |
| 17 | Yalfani et al  2024 | The effects of virtual reality training on postural sway and physical function performance on older women with chronic low back pain: A double-blind randomized clinical trial | Ineligible population |
| 18 | Rughani et al  2023 | The selfBACK artificial intelligence-based smartphone app can improve low back pain outcome even in patients with high levels of depression or stress | Ineligible population |
| 19 | Mcconnell et al  2024 | A multicenter feasibility randomized controlled trial using a virtual reality application of pain neuroscience education for adults with chronic low back pain | Ineligible population |
| 20 | Harvie et al  2024 | A randomized controlled pilot study examining immediate effects of embodying a virtual reality superhero in people with chronic low back pain | Ineligible intervention |
| 21 | Garcia et al  2021 | An 8-Week Self-Administered At-Home Behavioral Skills-Based Virtual Reality Program for Chronic Low Back Pain: Double-Blind, Randomized, Placebo-Controlled Trial Conducted During COVID-19 | Data unavailable / not extractable |
| 22 | Xiao et al  2025 | Clinical Efficacy of Multimodal Exercise Telerehabilitation Based on AI for Chronic Nonspecific Low Back Pain: Randomized Controlled Trial | Ineligible study design |
| 23 | Massah et al  2025 | Comparison of the Acute Effects of Virtual Reality Exergames and Core Stability Exercises on Cognitive Factors, Pain, and Fear Avoidance Beliefs in People with Chronic Nonspecific Low Back Pain | Ineligible intervention |
| 24 | Zheng et al  2022 | Does m-health-based exercise (guidance plus education) improve efficacy in patients with chronic low-back pain? A preliminary report on the intervention’s significance | Ineligible study design |
| 25 | Maddox et al  2024 | In-home virtual reality program for chronic low back pain: durability of a randomized, placebo-controlled clinical trial to 18 months post-treatment | Ineligible population |
| 26 | Yelvar et al  2017 | Is physiotherapy integrated virtual walking effective on pain, function, and kinesiophobia in patients with non-specific low-back pain? Randomised controlled trial | Ineligible intervention |
| 27 | Santos et al  2024 | Physical Activity Supported by Low-Cost Mobile Technology for Back Pain (PAT-Back) to Reduce Disability in Older Adults: Results of a Feasibility Study | Ineligible intervention |
| 28 | Li et al  2025 | The effectiveness of artificial intelligence health education accurately linking system on self-management in non-specific lower back pain patients | Ineligible population |
| 29 | Valenzuela-Pascual et al 2015 | The influence of a biopsychosocial educational internet-based intervention on pain, dysfunction, quality of life, and pain cognition in chronic low back pain patients in primary care: a mixed methods approach | Ineligible population |
| 30 | Shi et al  2024 | The Physical and Psychological Effects of Telerehabilitation-based Exercise for Patients with Non-specific Low Back Pain: A Prospective Randomized Controlled Trial | Data unavailable / not extractable |
| 31 | van der Veen et al  2026 | Virtual immersive gaming to optimize recovery (VIGOR) in low back pain: A phase II randomized controlled trial | Ineligible population |
| 32 | Spiegel et al  2026 | Randomized-controlled trial of skillsbased vr vs. distraction vr vs. sham VR for chronic low back pain | Data unavailable / not extractable |
| 33 | Day et al  2024 | The effects of telehealth-delivered mindfulness meditation, cognitive therapy, and behavioral activation for chronic low back pain: a randomized clinical trial | Ineligible intervention |
| 34 | Valenzuela-Pascual et al 2026 | Feasibility of Gamified Web-Based Pain Neuroscience Education for Chronic Low Back Pain | Ineligible intervention |
| 35 | Dosbaba et al  2025 | Comparison of hybrid guided home-based and outpatient rehabilitation in patients with chronic low back pain: A randomized controlled trial | Ineligible intervention |
| 36 | Garreta-Catala et al  2023 | Feasibility of a multidisciplinary group videoconferencing approach for chronic low back pain: a randomized, open-label, controlled, pilot clinical trial (EN-FORMA) | Ineligible intervention |
| 37 | Shirado et al  2010 | Multicenter randomized controlled trial to evaluate the effect of home-based exercise on patients with chronic low back pain: the Japan low back pain exercise therapy study | Ineligible intervention |
| 38 | Ben Salah Frih et al  2009 | Efficacy and treatment compliance of a home-based rehabilitation programme for chronic low back pain: a randomized, controlled study | Ineligible intervention |
| 39 | Zygouna et al  2025 | Comparison of the effectiveness of home-based video-supported McKenzie versus Pilates exercise programs in patients with chronic non-specific low back pain: a randomized controlled trial | Ineligible study design |
| 40 | Moreno-Ligero et al  2026 | Effectiveness of a m-Health-Delivered Home-based Exercise Program for Self-management of Chronic Low Back Pain: PainReApp Randomized Controlled Trial | Ineligible population |
| 41 | Cui et al  2023 | Randomized-controlled trial assessing a digital care program versus conventional physiotherapy for chronic low back pain | Ineligible population |
| 42 | Toelle et al  2019 | App-based multidisciplinary back pain treatment versus combined physiotherapy plus online education: a randomized controlled trial | Ineligible population |
