# Supplementary material for: Effectiveness of Telerehabilitation for Chronic Nonspecific Low Back Pain: Systematic Review and Meta-Analysis of Randomized Controlled Trials
Source: J Med Internet Res. 2026 Aug 10;28:e94835. doi: 10.2196/94835 (PMC13458355; doi:10.2196/94835)
Supplement: Supplementary file 4 [file jmir-v28-e94835-s004.docx]

**Figure S1. Subgroup analysis by delivery mode: pain intensity**

**Figure S2. Subgroup analysis by supervision level: pain intensity**

**Figure S3. Subgroup analysis by delivery mode: functional disability**

**Figure S4. Subgroup analysis by supervision level: functional disability**

**Figure S5. Subgroup analysis by delivery mode: kinesiophobia**
