# Supplementary material for: Effectiveness of Telerehabilitation for Chronic Nonspecific Low Back Pain: Systematic Review and Meta-Analysis of Randomized Controlled Trials
Source: J Med Internet Res. 2026 Aug 10;28:e94835. doi: 10.2196/94835 (PMC13458355; doi:10.2196/94835)
Supplement: Supplementary file 5 [file jmir-v28-e94835-s005.docx]

**Figure S1. Leave-one-out sensitivity analysis of pain intensity**

**Figure S2. Sensitivity analysis excluding small-sample studies for pain intensity**

**Figure S3. Sensitivity analysis excluding studies with high risk of bias for pain intensity**

**Figure S4. Leave-one-out sensitivity analysis of functional disability**

**Figure S5. Sensitivity analysis excluding studies with high risk of bias for functional disability**

**Figure S6. Leave-one-out sensitivity analysis of kinesiophobia**

**Figure S7. Sensitivity analysis excluding studies with high risk of bias for kinesiophobia**

**Figure S8. Funnel plot for pain intensity**

**Figure S9. Funnel plot for functional disability**
